# Biomimetic Citrate-Coated Luminescent Apatite Nanoplatforms for Diclofenac Delivery in Inflammatory Environments

**DOI:** 10.3390/nano12030562

**Published:** 2022-02-06

**Authors:** Sandra Maria Cano Plá, Annarita D’Urso, Jorge Fernando Fernández-Sánchez, Donato Colangelo, Duane Choquesillo-Lazarte, Riccardo Ferracini, Michela Bosetti, Maria Prat, Jaime Gómez-Morales

**Affiliations:** 1 Laboratorio de Estudios Cristalográficos, IACT, CSIC-UGR, Avda. Las Palmeras, n° 4, E-18100 Granada, Spain; sandra@lec.csic.es (S.M.C.P.); duane.choquesillo@csic.es (D.C.-L.); 2 Dipartimento di Scienze della Salute, Università del Piemonte Orientale, “A. Avogadro” Via Solaroli, 17, 28100 Novara, Italy; annarita.durso@med.uniupo.it (A.D.); donato.colangelo@med.uniupo.it (D.C.); 3 Department of Analytical Chemistry, Faculty of Sciences, University of Granada, Avda. Fuentenueva s/n, 18071 Granada, Spain; jffernan@ugr.es; 4 Dipartimento di Scienze Chirurgiche e Diagnostiche Integrate, Università di Genova, Viale Benedetto XV 6, 16132 Genova, Italy; ferracini@edu.unige.it; 5 Ospedale Koelliker, Corso Galileo Ferraris, 247/255, 10134 Torino, Italy; 6 Dipartimento di Scienze del Farmaco, Università del Piemonte Orientale “A. Avogadro”, Via Bovio 4, 28100 Novara, Italy; michela.bosetti@uniupo.it; 7 Centro di Biotecnologie per la Ricerca Medica Applicata (BRMA), Via Solaroli 17, 28100 Novara, Italy; 8 Consorzio Interuniversitario per Biotecnologie (CIB), Località Padriciano 99, 34149 Area di Ricerca, Italy; 9 Consorzio Interuniversitario Nazionale per la Scienza e Tecnologia dei Materiali (INSTM), Via Giuseppe Giusti, 9, 50121 Firenze, Italy

**Keywords:** inflammation treatment, diclofenac-loaded nanoparticles, apatite, Tb^3+^-doped apatite, luminescence, cytocompatibility

## Abstract

Luminescent nanoparticles are innovative tools for medicine, allowing the imaging of cells and tissues, and, at the same time, carrying and releasing different types of molecules. We explored and compared the loading/release ability of diclofenac (COX-2 antagonist), in both undoped- and luminescent Terbium^3+^ (Tb^3+^)-doped citrate-coated carbonated apatite nanoparticles at different temperatures (25, 37, 40 °C) and pHs (7.4, 5.2). The cytocompatibility was evaluated on two osteosarcoma cell lines and primary human osteoblasts. Biological effects of diclofenac-loaded-nanoparticles were monitored in an in vitro osteoblast’s cytokine–induced inflammation model by evaluating COX-2 mRNA expression and production of PGE_2_. Adsorption isotherms fitted the multilayer Langmuir-Freundlich model. The maximum adsorbed amounts at 37 °C were higher than at 25 °C, and particularly when using the Tb^3+^ -doped particles. Diclofenac-release efficiencies were higher at pH 5.2, a condition simulating a local inflammation. The luminescence properties of diclofenac-loaded Tb^3+^ -doped particles were affected by pH, being the relative luminescence intensity higher at pH 5.2 and the luminescence lifetime higher at pH 7.4, but not influenced either by the temperature or by the diclofenac-loaded amount. Both undoped and Tb^3+^-doped nanoparticles were cytocompatible. In addition, diclofenac release increased COX-2 mRNA expression and decreased PGE_2_ production in an in vitro inflammation model. These findings evidence the potential of these nanoparticles for osteo-localized delivery of anti-inflammatory drugs and the possibility to localize the inflammation, characterized by a decrease in pH, by changes in luminescence.

## 1. Introduction

Nanotechnology is finding increasing applications in medicine; in particular nanoparticles (NPs) can be used as vehicles to transport and deliver different classes of biologically active molecules (so-called nanocarriers), including chemotherapeutics, antibiotics, anti-inflammatory drugs, hormones, fluorophores, targeting agents [1,2,3,4,5,6,7], depending on the pathology considered, acting both in diagnosis and therapy. Their main advantage is linked to their nanoscale dimensions, enabling them to carry high amounts of molecules to chosen body sites, in the meantime protecting the drugs from rapid degradation or clearance. This allows to reduce the dose of the administered drugs, lowering or eliminating their unwanted systemic side effects. From the other side, also the nature/composition of the nanocarrier can vary depending on the diseases to be treated. Today bone tissue pathologies, such as osteoporosis, osteoarthritis and rheumatoid arthritis represent important health problems with considerable socio-economic burden, linked to the general population aging [8,9]. These musculo-skeletal disorders are characterized by a clinical condition of inflammation. In osteoporosis osteocatabolic processes prevail during bone remodelling, leading to a decrease of trabecular mass density, increasing the risk of bone fracture with concurrent inflammatory reaction [10]. In osteoarthritis the articular cartilages are consumed exposing the bones in the joint to mechanical stresses, with the development of a painful inflammatory reaction [11], while rheumatoid arthritis is an autoimmune disease characterized by chronic inflammation of synovial tissues, joints and cartilage leading to function loss and joint destruction [12]. For all these pathologies the available therapeutic strategies are still unsatisfactory, namely because of the associated side effects.

In this context, apatite (Ap) NPs, which consist of calcium phosphate and closely mimic bone apatite nanocrystals both from chemical and structural points of view, are particularly suited for therapeutic and diagnostic applications. Bone nanoapatite is nonstoichiometric calcium- (and OH-) deficient in respect to the mineral hydroxyapatite [HA, Ca_10_(PO_4_)_6_(OH)_2_], it incorporates substituting ions such as CO_3_^2−^, Mg^2+^, Na^+^ and other minor elements in its crystal structure [13], and contains citrate molecules strongly adsorbed on its surface [14]. Synthetic apatite-based materials including injectable calcium phosphates, or natural and synthetic polymer-apatite composites, are being used for bone repair applications exploiting their well-known properties of biodegradability, bioactivity and osteoinductivity, besides the capability of improving bone mechanical properties [15,16,17,18]. The apatite surfaces can be modified (charged to improve the interactions with living cells, particularly the osteoblasts) [19]. In addition, Ap NPs have already been shown to be valuable nanocarriers for different types of molecules [20], including nucleic acids [21], proteins [22], antibiotics [3], chemotherapeutics [23,24], fluorophores [25], and luminescent moieties [26,27]. Among their advantageous properties are high biocompatibility, good biodegradability, high loading capacity with the ability to bind moieties through both surface calcium and phosphate groups by isothermal adsorption. Moreover, because of their good stability at physiological pH with partial solubility at acidic pH they behave as smart complexes sensitive to local stimuli, e.g., binding drugs at physiological pH and releasing them at acidic pH [28], as the one found in inflamed or tumor tissues. Synthetic Ap NPs with CO_3_^2−^ substitutions and covered by a certain amount of citrate, makes them even more biomimetic to bone apatite in terms of chemical composition and reactivity [14,29]. Lanthanide elements such as Eu^3+^ and Tb^3+^ have been recently employed to prepare luminescent apatite-based nanomaterials for drug release and bioimaging applications [30,31]. They exhibit different fluorescence emission colors (red and green, respectively), long luminescence lifetimes, and good resistance to photobleaching. In addition to these features, Tb^3+^ was found to have bactericidal activity [32], and to promote adhesion and osteogenic differentiation of mesenchymal stem cells [33,34].

Sodium diclofenac (DF) is a non-steroidal anti-inflammatory drug (NSAID) with analgesic, anti-inflammatory and anti-pyretic activities, exerting its activity by competitively blocking cyclooxygenases-2 (COX-2) enzymatic activity responsible for the synthesis of inflammatory mediators, e.g., prostaglandin E_2_ (PGE_2_) [35,36]. As all the NSAIDs, DF has adverse systemic side effects, such as gastrointestinal ulceration and bleeding, hepato-renal dysfunction, disorders in the cardiovascular and central nervous systems, and skin reactions [37,38]. Local delivery of this drug via Ap NPs would offer a method of bypassing inconveniences. In addition, the use of Tb^3+^-doped Ap NPs would allow their localization by luminescence emission. Thus, our aim is to explore the loading/release behavior, luminescence properties, and in vitro biological effects of these NPs in normal physiological conditions and in a condition simulating inflammation.

In particular, herein we produced biomimetic citrate-coated CO_3_-Ap (cAp) and citrate-coated Tb^3+^-doped CO_3_-Ap (cAp-Tb) NPs. Next, we studied the adsorption and release of DF at physiological pH 7.4 (25 and 37 °C), and release at pHs 7.4 and 5.2 simulating local inflammation, as well as the luminescent properties of DF-loaded cAp-Tb NPs at pHs 7.4 and 5.2, and at 25, 37 and 40 °C. Then, we tested their cytocompatibility on different cell lines of bone origin, as well as on human primary osteoblasts (hOB) and osteoblasts differentiated in vitro from adipose-derived mesenchymal stem cells (differentiated hOB). Finally, we analysed the anti-inflammatory activity of DF-loaded cAp NPs on osteoblasts treated with a mixture of inflammatory cytokines (IL-1β, TNF-α, IFN-γ) by evaluating COX-2 activity and cellular production of PGE_2_.

## 2. Materials and Methods

### 2.1. Reagents

Diclofenac sodium salt (2-[(2,6-Dichlorophenyl)amino]benzeneacetic acid sodium salt, MW = 318.13), calcium chloride dihydrate (CaCl_2_·2H_2_O, Bioxtra, ≥99.0% pure, MW = 147.01), terbium (III) chloride anhydrous (TbCl_3_, 99.9% pure, trace metals, MW = 265.28), sodium citrate tribasic dihydrate (Na_3_(cit)·2H_2_O, cit = citrate = C_6_H_5_O_7_, ACS reagent, ≥99.0% pure; MW = 294.1), and sodium phosphate dibasic (Na_2_HPO_4_, ACS reagent, ≥99.0% pure, MW = 141.96) were provided by Sigma-Aldrich (St. Louis, MO, USA), while sodium carbonate monohydrate (Na_2_CO_3_·H_2_O, ACS reagent, 99.5% pure, MW = 124) and hydrochloric acid (HCl, ACS reagent, 37 wt % in H_2_O, MW = 36.46) were supplied by Merck (Darmstadt, Germany) and Panreac (Barcelona, Spain), respectively. The solutions were prepared with Milli-Q water (deionized 0.22 μS, 25 °C, Millipore, Burlington, MA, USA).

### 2.2. Preparation of cAp and cAp-Tb Nanoparticles

Nanoparticles of cAp were prepared by the method of thermal decomplexing of Ca^2+^/cit/phosphate/carbonate solutions [29]. A solution of composition 0.06 M Na_2_HPO_4_ + 0.1 M Na_2_CO_3_ (50 mL) was mixed with a solution of composition 0.1 M CaCl_2_ + 0.2 M Na_3_(cit) (50 mL) at 4 °C in a Pyrex glass bottle, and the pH adjusted to 8.5 with diluted HCl. The bottle was immediately introduced in a water bath at 80 °C and then in an oven at the same temperature for 96 h. For the preparation of cAp-Tb nanoparticles the second solution was composed of 0.010 M Tb^3+^ + 0.09 M CaCl_2_ + 0.2 M Na_3_(cit). This experiment lasted 4 h. Both precipitates were washed 4 consecutive times by centrifugation (9000 rpm, 30 min each) using Milli-Q water and dried in an oven with circulating forced air at 37.5 °C for 4 days.

### 2.3. Characterization of the Nanoparticles

The nanoparticles were characterized by X-ray diffraction (XRD) using a Bruker D8 Advance Vario Serie II (Bruker AXS, (Bruker GmbH, Karlsruhe, Germany) using CuKα radiation (1.5406 Å). Fourier Transform Infrared Spectrum (FTIR) was recorded with a Perkin-Elmer Spectrum One FTIR (Perkin Elmer, Shelton, WA, USA) in the wavenumber range from 4000 cm^−1^ a 400 cm^−1^. Plates were prepared with a concentration of ~ 1 wt% in anhydrous KBr (MW = 119) and then pressed with a hydraulic pump to 10 tons. Raman spectrum were recorded with a LabRAMHR spectrometer (Jobin-Yvon, Horiba, Tokyo, Japan) provided of a laser diode that emits at a wavelength of 532 nm. Transmission electron microscopy images (TEM) were taken with a Libra 120 Plus TEM instrument (EELS) at 80 kV (Carl Zeiss, Jena, Germany). Prior observation, the samples were dispersed in absolute ethanol (≥ 99.8% *v*/*v*) and deposited on copper microgrids coated with film of FORMVAR carbon. The particle size distribution (PSD) and ζ-potential were analysed by dynamic light scattering (DLS) with a Zetasizer Nano ZS analyser (Malvern Instruments Ltd., Malvern, UK) in aqueous suspensions (~0.5 mg/mL, room temperature) contained in polystyrene vials. For measurements of ζ-potential versus pH, suspensions of the nanoparticles were prepared at pHs from 4 to 9 using the MPT2 autotitrator with dilute HCl and NaOH (MW 39.997) solutions (0.25 and 0.1 M, respectively). Elemental analysis of Tb^3+^ was carried out by inductively coupled plasma mass spectroscopy (ICP-MS) using a Perkin Elmer NexION 300D ICP Mass spectrometer (Perkin Elmer, Beaconsfield, UK). C and H were determined by thermoanalysis using Thermo Scientific™ FLASH 2000 CHNS/O Analyzer of Thermo Fisher Scientific (Waltham, MA, USA).

### 2.4. Adsorption Kinetics

For adsorption kinetic studies we prepared 7 eppendorf tubes containing 2 mg of adsorbent (cAp and cAp-Tb) in 1 mL of phosphate buffered saline (PBS) solution containing DF at the maximum solubility (0.45 mg/mL). The PBS buffer (pH = 7.4) was prepared with the following composition: 137 mM NaCl (MW = 58.44), 2.7 mM KCl (MW = 74.55), 10 mM Na_2_HPO_4_ and 1.8 mM KH_2_PO_4_ (MW = 136.08). The tubes were left in the dark each one for a different time: 1 h, 2 h, 4 h, 8 h, 15 h, 24h and 48 h. At the end of the experiment, the solid was decanted by centrifugation at 10,000 rpm for 5 min. The absorbance of the residual solution was then measured with an UV-Vis (Agilent Technologies Cary Series UV-Vis, Agilent Technologies, Madrid, Spain) Spectrophotometer at λ = 280 nm [39] and the amount of DF adsorbed per unit mass of adsorbent as a function of time determined. The DF concentration *C_DF_* was determined from the calibration straight line whose equation is the following:

*Abs* = 32.267*C_DF_* + 0.02 (1)

### 2.5. Adsorption Isotherms

Experiments were carried out in several Eppendorf tubes containing 2 mg of adsorbent (cAp and cAp-Tb) in 1 mL of PBS solution with varying concentrations of DF (0.05, 0.1, 0.15, 0.20, 0.25, 0.30, 0.35, 0.40 and 0.45 mg/mL). The trials were carried out in triplicate under continuous stirring at 25 and 37 °C for 24 h (time higher than that necessary to reach the adsorption equilibrium according to adsorption kinetics). At the end, samples were centrifuged at 10,000 rpm for 5 min, and then filtered. The equilibrium concentration (*C_eq_*, mg/mL) of DF in the supernatants were analysed by UV-Vis applying Equation (1). The adsorbed amount of DF per unit mass of adsorbent (*Q_ads_*, mg/mg) was then calculated by difference between the initial (*C_0_*) and equilibrium (*C_eq_*) concentrations, divided by the mass (mg) of adsorbent, for a 1 mL total volume. The plot of *Q_ads_* versus *C_e_* is the adsorption isotherm.

### 2.6. Release Experiments

Release experiments at a function of time (1 h to 7 days) were performed at 25 and 37 °C under agitation using PBS (pH 7.4) and citrate/NaOH (pH = 5.2) buffers solutions. Each Eppendorf tube contained 2 mg of nanoparticles (DF-cAp and DF-cAp-Tb) with maximum adsorbed DF amount (*Q*_max_) immersed in 1 mL buffer solution. At each programmed time, the suspensions were centrifuged at 10,000 rpm for 5 min, filtered, and the *C_DF_* in the supernatant analysed by UV-Vis. All assays were performed in triplicate and in the dark since the DF is photosensitive.

### 2.7. Luminescence Studies of cAp-Tb-DF Samples

Measurements were carried out at 25, 37, and 40 °C, at pHs 5.2 and 7.4 (adjusted with diluted HCl and NaOH), using a 0.5 mg/mL suspension of cAp-Tb sample. The nanoparticles were loaded in 0.0, 0.1, 0.3 and 0.4 mg/mL DF solution for 24 h, as in the adsorption experiments. The excitation and emission wavelengths used were λ_exc_ = 350 nm and λ_em_ = 545 nm. The instrumental parameters for the spectral characterization of the particles in aqueous suspensions were: t_d_ = 120 µs, t_g_ = 5 ms, slitswidth_exc/em_ = 20/20 nm and detector voltage 750 v. The instrumental parameters for the lifetime characterization were: t_d_ = 120 µs, t_g_ = 0.01 ms, slitswidth_exc/em_ = 20/20 nm and detector voltage 900 V.

### 2.8. Cells

The two human osteosarcoma cell lines MG-63 (CRL-1427) and U-2 OS (HTB-96TM), which were obtained from ATTC, were grown in Dulbecco modified Eagle’s medium (DMEM) (Sigma-Aldrich, Milan, Italy) supplemented with 10% foetal bovine serum (FBS), antibiotic solution (streptomycin 100 µg/mL and penicillin 100 U/mL, Sigma-Aldrich) and 2 mM L-glutamine (complete medium) in a humidified atmosphere containing 5% CO_2_ at 37 °C. Human primary osteoblasts (hOB) at passages from 2 to 7 obtained from explants of human trabecular bone fragments from knee joints taken at surgery (kindly provided by the Orthopedic Institute, Major Hospital Charity, Novara, Italy) were cultured in Iscove’s modified Dulbecco’s medium supplemented as above (complete Ob medium), as described previously [40]. Mesenchymal Stem Cells (MSCs) were obtained from the stromal vascular fraction of lipoaspirates, after enzymatic digestion with Collagenase NB4 (SERVA Electrophoresis, GmbH, Heidelberg, Germany) and elimination of blood cells, isolated and characterized as described by Roato [41]. MSCs were induced to osteogenesis by 2 week treatment with (complete Ob medium containing 50 mg/mL ascorbic acid (MW 176.12), 10 mM β-glicerophosphate (MW 216.04), and 10 nM dexamethasone (MW 392.5) (all from Sigma-Aldrich), which were changed every 3 days [42]. The osteogenic differentiation was evaluated by staining with an alkaline phosphatase detection kit (Millipore, Merk Millipore, Milano, Italy) according to the manufacturer’s protocol (see Supplementary data). These cells were named differentiated hOB. All patients were free of systemic disease or treatment. The samples represent surgical discharge materials, and therefore their use does not need ethics committee approval. All patients were informed about the scientific use of the materials removed and gave their consent.

### 2.9. Cytocompatibility Tests

MG-63 and U-2OS cells (5000 cells/wells in 96-well plates) were seeded and 24 h after different concentrations (ranging from 0.1 to 100 µg/mL) of the differentially DF uploaded nanoparticles, either Tb-doped or undoped, were added in 100 µL of fresh medium. Hydrogen peroxide (1 µM) was used as control of toxicity. After 72 h incubation, cell viability was evaluated by the 3-(4,5-Dimethylthiazol-2-yl)-2,5-diphenyltetrazolium bromide) (MTT, Sigma) colorimetric assay, and the optical density was measured in a multiwell reader (2030 Multilabel Reader Victor TM X4, Perkin Elmer, Beaconsfield, UK) at 570 nm, as described [23]. In the case of hOB and differentiated hOB (15,000 cells/wells in 96-well plates) the assay was continued for 7 days, with careful medium changes every 3–4 days. Viability of parallel cultures of untreated cells was used as 100% viability, and values obtained from cells undergoing the different treatments were referred to this value. Experiments were performed 3 times using 3 replicates for each sample.

### 2.10. Inflammatory Cytokine Treatment

hOS, both induced and not, were seeded at a concentration of 15,000 cells/microwell and when 95% confluent (after about 3–4 days) recombinant human IL-1β, human TNF-α and human IFN-γ (ImmunoTools GmbH, Friesoythe, Germany) were added in complete culture medium at final concentrations of 1, 10, 100 ng/mL, respectively (cytokine mix), following a published protocol with some modifications [43]. The following day medium was changed with all cytokine concentrations reduced to 1:4 and after a further day the medium with the same cytokine concentration was changed and 50 µg/mL of UV-sterilized Tb-doped NPs functionalized with DF or not and comparable amounts of soluble DF were added. Medium was then changed on day 3, maintaining the same concentrations of cytokines and of the different NPs and controls. Some experiments lasted 1 or 5 days.

### 2.11. Quantitative Real-Time PCR (qPCR)

hOS (500,000/6 cm diameter plate) were seeded and when confluent they were treated for induction of inflammation by the addition of the cytokines mixture. After 24 h cAp-Tb uploaded with DF or not were added at a concentration of 50 µg/mL. In this case, 16 h later, total cell RNAs were extracted with Trizol (Invitrogen Life Technologies, Monza, Italy). After RNA purification and treatment with DNAse I (Fermentas, St. Leon-Rot, Germany), 1 μg was retrotranscribed in cDNA with the RevertAid™ H Minus First Strand cDNA Synthesis Kit (Fermentas) using oligo(dT) primers. Gene assays were performed in triplicate for each treatment in a 20 μL reaction volume containing 1 μL of RT products, 10 μL Sso-Fast EVA Green SMX (Bio-Rad, Hercules, CA, USA), 500 nM each forward and reverse primers (COX-2, Fw: TATCACAGGCTTCCATTGACC; Rev: TTTCTACCAGAAGGGCAGGAT) [44]. Gene expression was normalized on the housekeeping gene ribosomal 18S rRNA (Fw: 5′-GTGGAGCGATTTGTCTGGTT-3′; Rev: 5′-ACGCTGAGCCAGTCAGTGTA-3′). Automated CFX96 real-time thermocycler (Bio-Rad) was used and the reaction conditions were 95 °C for 1 min, followed by 45 cycles at 98 °C for 5 s and anneal/extend step for 5 s at 60 °C, with data collection. At the end of these cycles, a melting curve (65 °C to 95 °C, with plate read every 0.5 °C) was performed to assess the specificity of the amplification product by single peak melting temperature verification. Results were analysed with Bio-Rad CFX Manager. Calculations and statistical analyses were performed using GraphPad Prism version 5.00 for Windows (GraphPad Software, San Diego, CA, USA).

### 2.12. PGE_2_ Production

PGE_2_ produced by cells and released in the culture medium was evaluated in an ELISA Kit (Cayman kit, Vinci-Biochem, Vinci, Italy), according to the manufacturer’s protocol. This is a competitive-binding assay, which was performed on 1/5 diluted samples in triplicates and was repeated twice. Optical absorbance was read at 405 nm.

### 2.13. Statistical Analysis

Data were statistically analysed and are expressed as mean ± standard deviation. Statistical analyses were performed using a one-way ANOVA with Bonferroni’s post test for grouped analyses using GraphPad Prism version 5.04 for Windows, GraphPad Software (GraphPad Prism, San Diego, CA, USA). Differences at *p* < 0.05 were considered to be statistically significant.

## 3. Results

### 3.1. Characterization of the NPs

**Figure 1:**
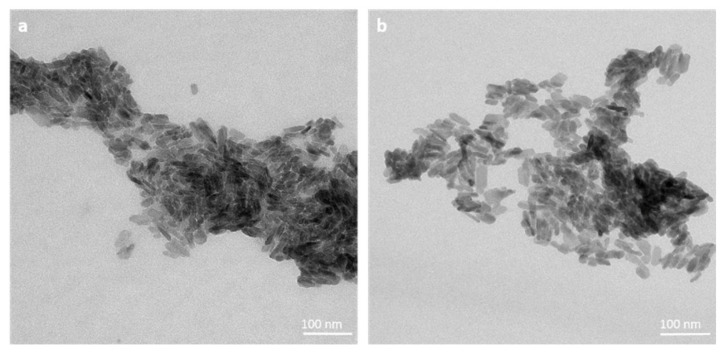
TEM micrographs of cAp (**a**) and cAp-Tb NPs (**b**) prepared by thermal decomplexing method [27,29].

Precipitation experiments yielded cAp and cAp-Tb NPs with needle-like morphologies, elongated along the *c*-axis, of 40 ± 10 nm and 30 ± 8 nm, respectively (Figure 1). XRD patterns (see Supplementary Materials, Figure S1) show the diffraction peaks at 2θ = 25.87° (002), 31.77°, 32.19° and 32.90° (planes (211), (112) and (300)), respectively, at 33.9° (202) and 39.81° (310) and minor reflections within the range 38°–55°, characteristics of the apatite phase (PDF 01-1008) [29].

FTIR spectra (Figure S2a) display the main band at 1000–1100 cm^−1^ (asymmetric stretching υ_3_PO_4_), the shoulder at ~965 cm^−^^1^ (symmetric stretching υ_1_PO_4_), and the less intense bands at ~608 and 568 cm^−^^1^ (bending mode υ_4_ PO_4_). The presence of carbonate (CO_3_^2−^) is attested by the small band at 873 cm^−^^1^ (υ_2_ CO_3_) and υ_3_CO_3_ mode, with bands ~1417 cm^−^^1^ and 1468 cm^−1^. The band at ~1590 cm^−1^ observed in both samples, is ascribed to the antisymmetric stretching frequencies of the carboxylate groups of the citrate, indicating the citrate molecules are adsorbed on the apatite surface, as previously reported [27]. On the other hand, the complementary characterization by Raman (Figure S2b) reveals the main band at 960 cm^−1^ which corresponds to the antisymmetric vibration mode of phosphate groups (υ_1_PO_4_) of the apatite phase [45]. The elemental composition of the new cAp-Tb sample revealed by ICP-MS and thermoanalysis yields 30.78 wt% Ca, 14.44 wt% P, 0.69 wt% Tb, 2.23 wt% C, and 0.53 wt% H.

The measurements of DLS of the dispersed nanoparticles in aqueous media are shown in Figure S3, which displays the plots as a) PSD in volume and b) cumulative volume oversize distribution. The last one shows D_10_ percentiles for cAp and cAp-Tb of 55 and 75 nm, respectively, which are close to the individual particle size observed by TEM. The D_50_ percentiles (75 and 276 nm) are affected by aggregation, especially in the cAp-Tb sample. The D_90_ are highly affected by aggregation.

### 3.2. Adsorption Isotherms

The evolution of the adsorbed amount of DF per unit mass of adsorbent (*Q*_ads_) with the time on both substrates (Figure S4) draws different profiles but reveals that adsorption equilibrium is reached after about 15 h. Thus, the adsorption experiments for the elaboration of the isotherms were performed at 24 h, to assure that all points were determined after reaching the equilibrium.

**Figure 2:**
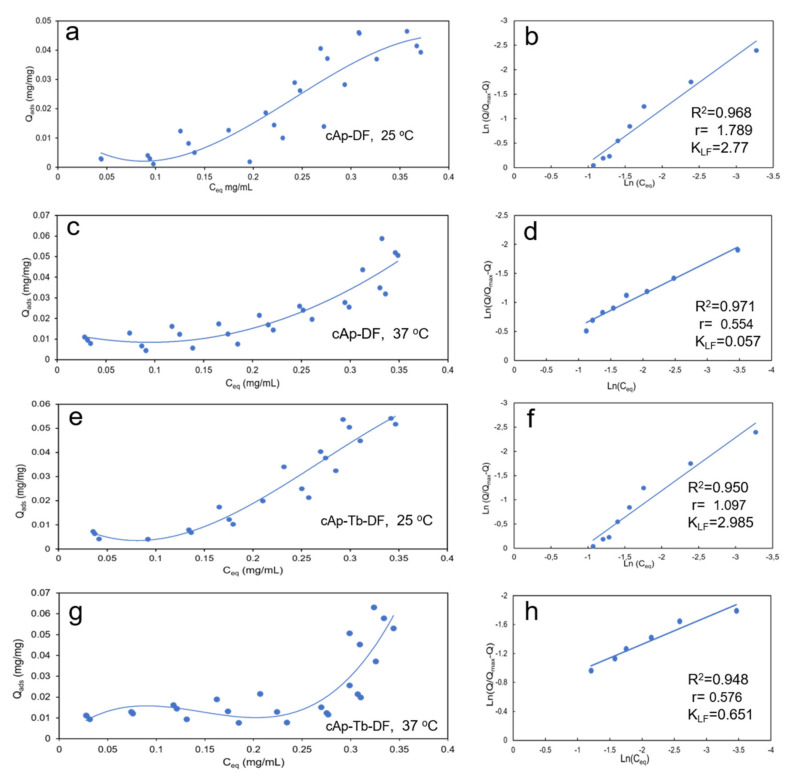
Adsorption isotherms of DF in (**a**) cAp at 25 °C, (**c**) cAp at 37 °C, (**e**) cAp-Tb at 25 °C, and (**g**) cAp-Tb at 37 °C. Fitting of adsorption data to the Langmuir-Freundlich model (**b**) cAp at 25 °C, (**d**) cAp at 37 °C, (**f**) cAp-Tb at 25 °C, and (**h**) cAp-Tb at 37 °C.

Figure 2 shows the adsorption isotherms (*Q_ads_* vs. *C_eq_*) of DF on cAp at 25 °C (a) and 37 °C (c), and on cAp-Tb at 25 °C (e) and 37 °C (g). Adsorption data were fitted to different adsorption models: Langmuir [44,45,46,47,48], Freundlich [49] and Langmuir-Freundlich [30,50,51]. The Langmuir model considers a monolayer of adsorbate and the surface of the adsorbent energetically homogeneous. The linearized equation is described as:

(2) $$1/Q_{eq}=1/Q_{max}+1/\left(Q_{max}\;K_{L}\;C_{eq}\;\right)$$

being $Q_{max}$ the maximum adsorbed amount per unit mass of adsorbent (mg/mg) and *K_L_* the Langmuir affinity constant (L/mg). The Freundlich model considers an energetically heterogeneous surface and multilayer adsorption. The linearized equation is:

(3) $$\log Q_{eq}=\log K_{F}+1/n\;\;\log C_{eq}$$

where the Freundlich constant $K_{F}$ and 1/*n* are related to the adsorption capacity of the substrate and the intensity of the adsorption, respectively. The third model, Langmuir- Freundlich, is more versatile, and simulate both behaviours. The linearized equation is the following:

(4) $$\ln\left(Q/\left(Q_{max}-Q\right)\right)=r\ln K_{LF}+r\ln C_{eq}$$

In equation 4 *K_LF_* is the Langmuir-Freundlich constant and *r* the cooperativity coeficient, with *r* > 1 indicating a positive cooperativity or *r* < 1 negative cooperativity [50].

Only the Langmuir-Freundlich model fitted reasonably the adsorption data (Figure 2b,d,f,h), with regression coefficients (R^2^) higher than 0.97 and 0.95 for cAp and cAp-Tb experiments, respectively, whereas the other two models yielded R^2^ < 0.7. This indicates that DF adsorbs in multilayers with a limited number of layers. The *K_LF_*,*r* and *Q_max_* values obtained by this model are shown in Table 1.

We can appreciate that at 25 °C the *K_LF_* values for cAp and cAp-Tb are higher than at 37 °C, revealing the higher affinity of DF molecules for the adsorbent at this temperature. In addition, the *r* values at 25 °C are higher than 1, while at 37 °C are lower, indicating the process is cooperative at 25 °C and non-cooperative at 37 °C. However, *Q_max_* is always higher in the cAp-Tb substrate irrespective of the temperature.

### 3.3. ζ-Potential versus pH of Unloaded and DF-Loaded cAp and cAp-Tb NPs Suspensions

**Figure 3:**
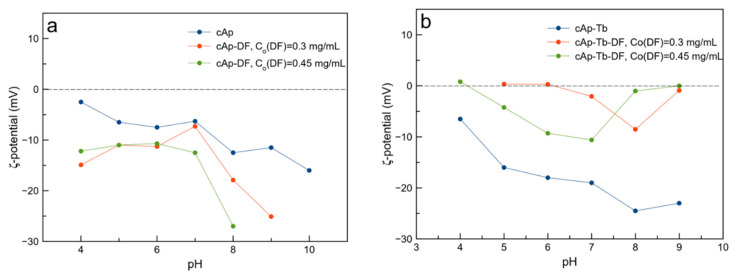
Plots ζ-potential versus pH: (**a**) cAp (blue), cAp-DF loaded in solutions of DF 0.3 mg/mL (red) (*Q_ads_* = 0.02304 mg/mg) and cAp-DF loaded in solutions of DF 0.45 mg/mL (green) (*Q_ads_* =0.04246 mg/mg). (**b**) cAp-Tb (blue), cAp-Tb-DF loaded in solutions of DF 0.3 mg/mL (red) (*Q_ads_* =0.02680 mg/mg) and cAp-Tb-DF loaded in solutions of DF 0.45 mg/mL (green) (*Q_ads_* =0.04435 mg/mg).

The measurements of ζ-potential of both type of unloaded NPs (blue lines in Figure 3) reveal a decrease to more negative values with increasing the pH, especially for the sample cAp-Tb, with values of −19 mV (pH 7) and of −16 mV (pH 5). This indicates that NPs tend to be dispersed in aqueous media at physiological and acidic pHs, which is a desirable feature in view of their applications in nanomedicine.

However, when loading the nanoparticles with DF in solutions of concentrations 0.3 and 0.45 mg/mL, we found a different behaviour depending on the type of adsorbent. Thus, when loading cAp, the ζ-potential of cAp-DF nanoassemblies decreases (more negative), especially when their payload is higher. The behaviour is the opposite when loading cAp-Tb nanoparticles, being the ζ-potential of the cAp-Tb-DF nanoassemblies higher (less negative), especially when they were loaded in the solution with the lower DF concentration at pH ≤ 7. This finding reveals a higher tendency of the cAp-Tb-DF nanoassemblies to aggregate. Nevertheless, for the cAp-Tb-DF sample (*C_o_* = 0.45mg/mL DF, *Q_ads_* = 0.04435 mg/mg), the ζ-potentials are still negative being −4.2 mV at pHs 5 and −10.6 mV at pH 7.

### 3.4. Release Profiles and Release Efficiency

**Figure 4:**
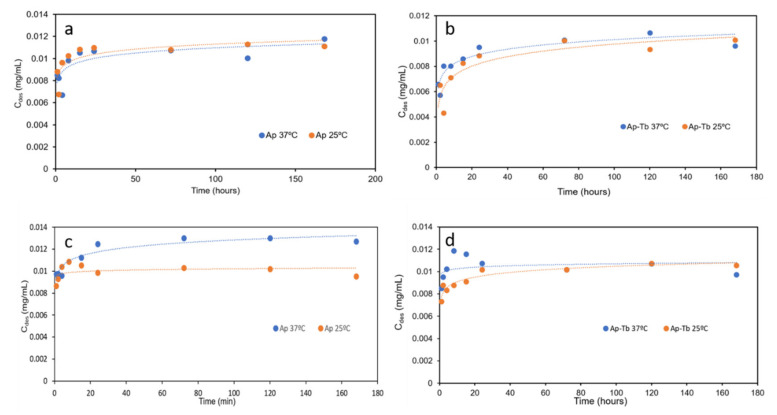
Release profiles of DF from (**a**) cAp-DF at 25 and 37 °C, pH 7.4; (**b**) cAp-Tb-DF at 25 and 37 °C, pH 7.4; (**c**) cAp-DF at 25 and 37 °C, pH 5.2, and (**d**) cAp-Tb-DF at 25 and 37 °C, pH 5.2.

Figure 4 shows the DF release profiles (*C_des_* vs. time) at pH 7.4 (a,b) and 5.2 (c,d) by comparing the effect of the temperature (25 and 37 °C) for both types of nanoparticles. It is observed that at pH 7.4 the released DF mass per mL of buffer solution at 37 °C is slightly higher than at 25 °C, and always higher for cAp than from cAp-Tb. At pH 5.2 the released amount from cAp-DF at 37 °C even increases within the time interval of the experiment (Figure 4c). It is worth mentioning the peculiar release profile of the cAp-Tb substrate at 37 °C, which shows a burst during the first 10 h, and then stabilizing with time.

**Figure 5:**
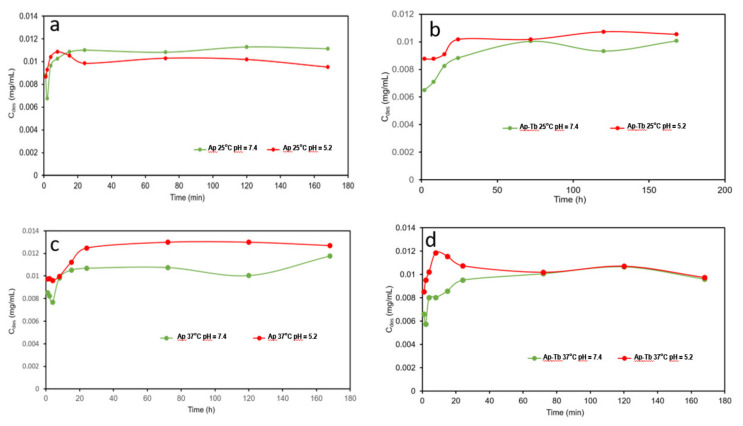
Release kinetics of DF from cAp-DF at 25 °C (**a**) and 37 °C (**c**) and from cAp-Tb-DF at 25 °C (**b**) and at 37 °C (**d**) at pH 7.4 (green line) and 5.2 (red line).

When comparing the effect of pH (7.4 and 5.2) on the release of DF a higher release was observed at pH 5.2 (citrate buffer) than at pH 7.4 (PBS buffer), at both temperatures, with the only exception for cAp at 25 °C after the first 10 h of the experiment, when the delivery trend versus pH is the opposite (Figure 5a–d). This pH-responsive drug release behaviour is very useful concerning DF delivery in pathological environments.

The release efficiency *Dr* [30,50] defined as the percentage of drug released at a given time (*t*) respect to maximum amount of drug adsorbed (Equation (5)),

(5) $$\;Dr\;\left(\%wt\right)=Q_{t}/Q_{max}\;\times 100$$

revealed that considering the larger times of the experiment, *Dr* and *Q_D,max_* for cAp-DF are in general larger than for cAp-Tb-DF at both pHs (see Table 2). This trend is just the opposite to that found in the adsorption experiments, in which the maximum adsorption corresponded to cAp-Tb.

### 3.5. Luminescence Properties of Unloaded and DF-Loaded cAp and cAp-Tb NPs Suspensions

It is well-known that Tb^3+^- luminescent chelates shows narrow-banded, line-type fluorescence with long Stokes shifts and high luminescence decay times [52]. This luminescent emission can be used for sensitized fluorescence detection of chelates [53], the development of FRET analytical methods [54], as well as for the incorporation of luminescent properties to materials in order to develop new imaging applications [29,34]. In this work, Tb^3+^ was incorporated to provide the nanocarrier with a luminescence signal which can be measured to determine where the particles are located.

**Figure 6:**
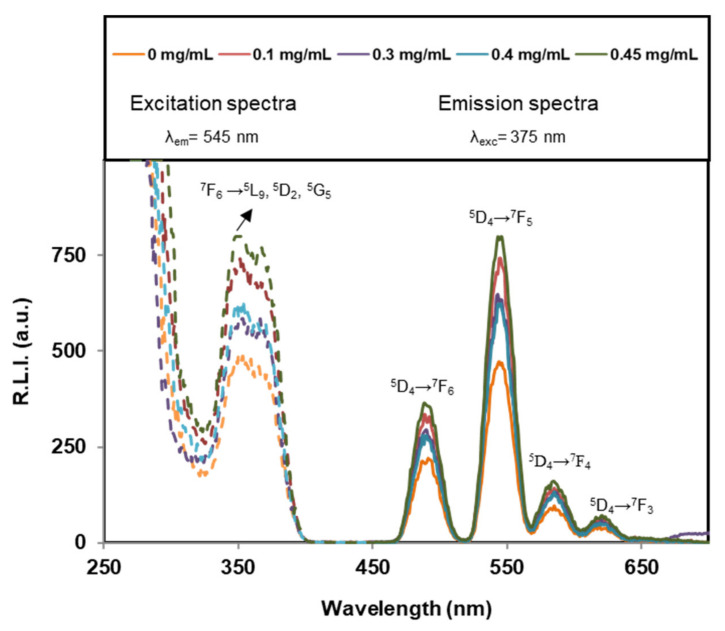
Excitation (dashed lines) and emission (solid lines) uncorrected spectra of cAp-Tb-DF samples containing different DF adsorbed amounts, dispersed in a pH 5.2 aqueous suspension at 0.5 mg/mL and 37 °C. The instrumental conditions were t_d_ = 120 µs, t_g_ = 5 ms, slitwidth_exc/em_ = 20 nm/20 nm and detector voltage 750 V.

Figure 6 shows the excitation and emission spectra of NPs dispersed in aqueous media at pH 5.2 at 37 °C which are similar to those obtained for 25 and 40 °C as well as for pH 7.4 at these three temperatures (see Supplementary Materials Figures S5–S9).

Concerning the excitation wavelength (λ_exc_), these particles can be excited at the Charge Tranfer Band, CTB [55], (centred at 230 nm approximately) obtaining the same emission spectra as that obtained by exciting at 350 nm [34]. In order to increase the biological applicability of the system, 350 nm (corresponding to the Tb^3+ 7^F_6_→^5^L_9_,^5^D_2_,^5^G_5_ transition [56] was selected as excitation wavelength.

Concerning the emission wavelengths (λ_em_), they are centred at 491, 545, 586 and 623 nm, corresponding to the Tb^3+ 5^D_4_→^7^F_6_, ^5^D_4_→^7^F_5_, ^5^D_4_→^7^F_4_ and ^5^D_4_→^7^F_3_ transitions, respectively [57]. The emission wavelength which produces the highest relative luminescence intensity (R.L.I.) corresponds to the hypersensitive transition without inversion centre (^5^D_4_→^7^F_5_, 545 nm for Tb^3+^) Therefore, the optimum λ_exc_ and λ_em_ of solid particles were 350 and 545 nm, respectively.

**Figure 7:**
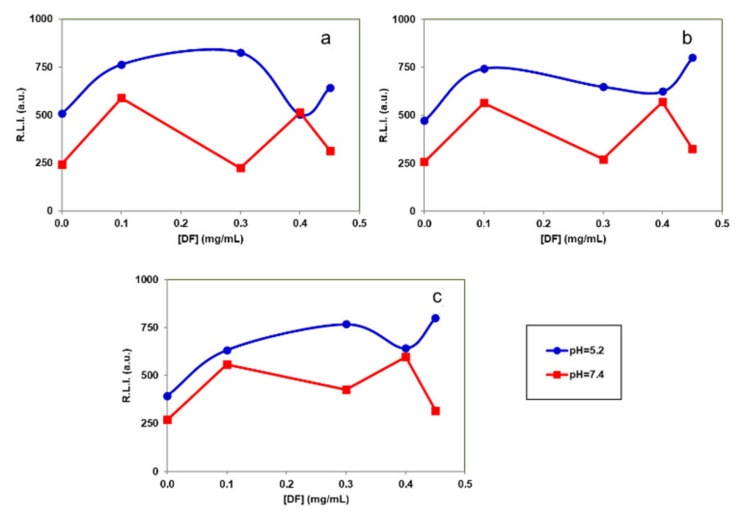
Effect of the DF concentration on the R.L.I. of the particles suspended in aqueous suspension at different pHs and temperatures: (**a**) T = 25 °C, (**b**) T = 37 °C and (**c**) T = 40 °C. Excitation (dashed lines) and emission (solid lines) uncorrected spectra of samples containing different DF concentrations dispersed in a pH 5.2 aqueous suspension at 0.5 mg/mL and 37 °C. The instrumental conditions were λ_exc/em_ = 350/545 nm, t_d_ = 120 µs, t_g_ = 5 ms, slitwidth_exc/em_ = 20/20 nm and detector voltage 750 V.

Figure 7 shows the effect of DF concentration. It is possible to conclude that the adsorption of DF on the particles does not affect significantly the Tb^3+^ luminescence emission. Therefore, this signal cannot be used to determine if DF is released or not.

**Figure 8:**
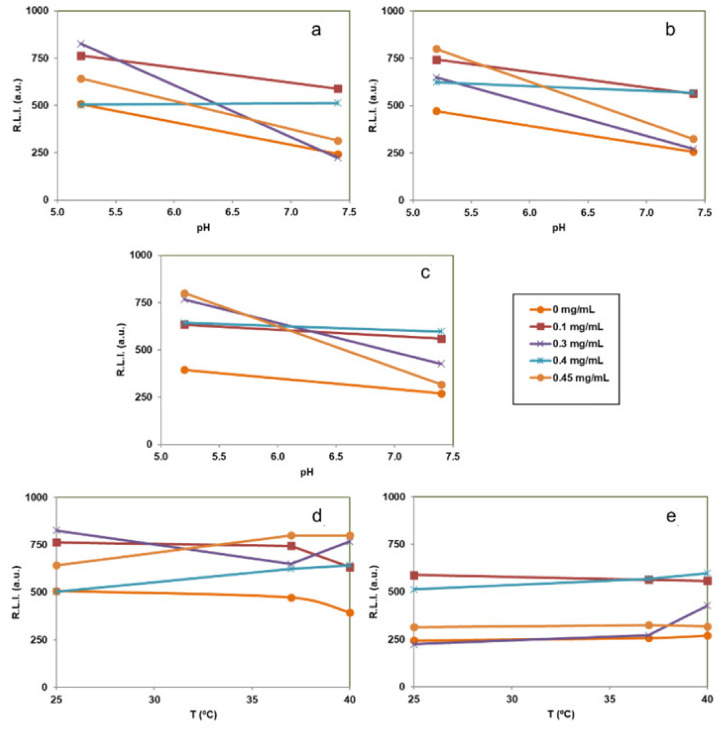
Effect of the pH and the temperature on the R.L.I. of the cAp-Tb-DF with different amounts of DF adsorbed suspended in aqueous media at different pHs and temperatures [(**a**) 25 °C, (**b**) 37 °C and (**c**) 40 °C, (**d**) pH 5.2 and (**e**) pH 7.4]. In all measurements the concentration of the suspended particles was 0.5 mg/mL and the instrumental conditions were λ_exc/em_ = 350/545 nm, t_d_ = 120 µs, t_g_ = 5 ms, slitwidth_exc/em_ = 20/20 nm and detector voltage 750 V.

Figure 8 shows the effect of pH and temperature. We can observe that, at all tested DF concentrations, the signals at pH 5.2 are higher than those obtained at pH 7.4. Thus, the luminescence emission of these particles could be used to determine pH changes, which are related to inflammatory processes. In addition, it is possible to conclude that the luminescence signal is not affected between 25 and 40 °C demonstrating that it can be used for biological applications, since the luminescence is not affected by local increases of temperature due to inflammatory processes.

Figures S10–S15 in Supplementary Materials show the luminescence decay curves. For each case, the decay profile was analysed as a single exponential component (Equation 6)

(6) $$R.L.I.=e^{\left(-\frac{t}{\tau }\right)\;}+C$$

**Figure 9:**
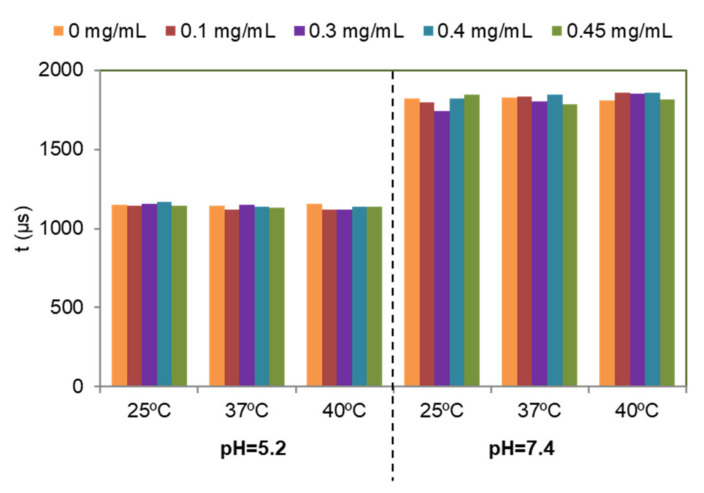
Effect of DF concentration, pH and temperature on the luminescence lifetime of the particles suspended in aqueous suspension. In all the cases the concentration of the suspended particles was 0.5 mg/mL and the instrumental conditions were λ_exc/em_ = 350/545 nm, t_d_ = 120 µs, t_g_ = 0.01 ms, slitwidth_exc/em_ = 20/20 nm and detector voltage 900 V.

Figure 9 shows the variation of the luminescence lifetime (τ) versus DF concentration, pH and temperature. It is shown that only pH is affecting the lifetime; larger lifetimes are obtained at pH 7.4 than at pH 5.2 while different DF concentrations and temperatures do not affect the luminescence lifetimes.

### 3.6. Cytocompatibility of the Different Nanoparticles

**Figure 10:**
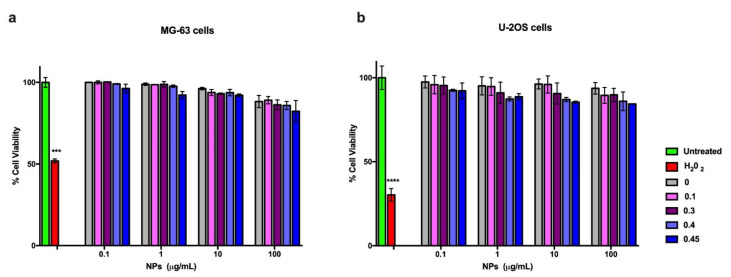
Viability of MG-63 cells (**a**) and of U-2OS cells (**b**) incubated with cAp-DF particles loaded with different DF concentration, ranging from 0.1 to 0.45 mg/mg NP, for three days. Viability was assessed in MTT assays. Data represent means ± sd of three independent experiments performed in triplicate and statistical analyses were carried on using One-way ANOVA, with Bonferroni comparison test. For statistical analysis all data were compared to untreated samples and only samples treated with 1µM H_2_O_2_ displayed statistically significant difference (*** *p* < 0.001, **** *p* < 0.0005).

**Figure 11:**
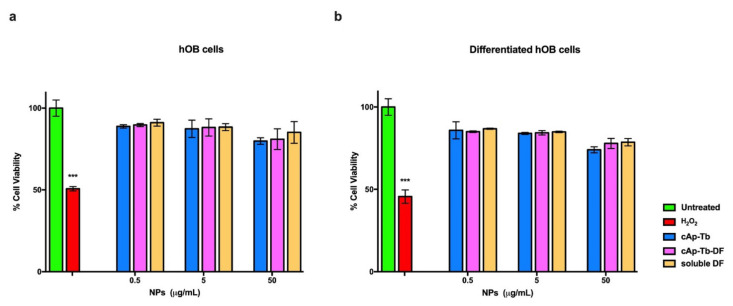
Viability of human primary osteoblasts hOB (**a**) and of hOB differentiated from mesenchymal stem cells (**b**) incubated with different concentrations of cAp-Tb particles loaded with DF (50 µg/mL) and unloaded, and comparable soluble DF amounts for seven days. Viability was assessed in MTT assays. Data represent means ± sd of three independent experiments performed in triplicate and statistical analyses were carried on using One-way ANOVA, with Bonferroni comparison test. For statistical analysis all data were compared to untreated samples and only samples treated with 1µM H_2_O_2_ displayed statistically significant difference (*** *p* < 0.001).

The cytocompatibility of cAp NPs, doped with Tb or not, and uploaded with different concentrations of DF was first assessed on two human osteosarcoma cell lines (MG-63 and U2OS) in MTT assays performed after 3 days of incubation with NPs (Figure 10 and Figure S16). No significant toxicity was observed in any condition; only when cells were incubated with the highest NPs concentration of 100 µg/mL cell viability was decreased, but it was always higher than 80%, which are values above the cut off of 70% indicated by ISO 10993-5:2009 [58]. cAp NPs cytocompatibility was tested also on primary human osteoblasts (hOB) and on differentiated hOBs, which stained positive for alkaline phosphatase (Figure S17), a typical marker of bone differentiation. In view of the fact that experiments of inflammation would last longer than 3 days, and that 100 µg/mL reduced somehow the viability of the osteosarcoma cell lines, MTT assays were carried out also after 1 week of incubation with NPs and lower doses of NPs, ranging from 50 to 0.5 µg/mL were assessed. Similar data were obtained at the two end-points evidencing no significant reduction in cell viability and results were in line with those obtained with the two osteosarcoma cell lines. In particular Figure 11 shows the results obtained with Tb-doped NPs. All the cells were sensitive to the addition of 1 µM hydrogen peroxide, since their viability was reduced to about 30–50%. Thus, these data show the good cytocompatibility of the different nanoparticles that will be used in this study.

### 3.7. Effect of DF-Loaded Nanoparticles on the Osteoblasts Treated for Inflammation: COX-2 Expression

**Figure 12:**
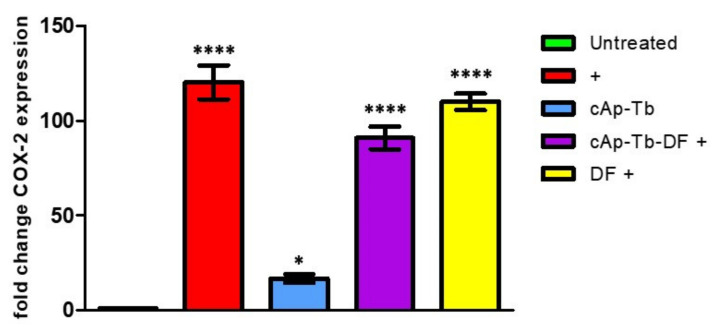
Effect of inflammation stimuli, cAp-Tb and soluble DF or cAp-Tb-DF, on COX-2 mRNA levels were assayed on hOB differentiated from mesenchymal stem cells. Cells were incubated for 24 h in presence or absence of the inflammatory stimuli. Data were calculated by comparing the fold increase vs. the untreated control and significance is shown on the figure. Bonferroni comparison post-test analysis of each treatment was calculated vs. the inflammation stimuli effects (marked as +). Data indicated that DF and cAp-Tb-DF did not significantly modify the expression pattern induced by inflammation. Data represent means ± SD of four independent experiments (* *p* < 0.01, **** *p* < 0.0005).

To assess the expression of COX-2 mRNA we performed semiquantitative RT-PCR. Differentiated hOB cells showed a low basal expression of the enzyme that was significantly increased after the stimulation with inflammation cytokines for 16 h, as determined by the calculation of relative expression (2^ –*ΔΔ*Ct; Figure 12). The exposure to cAp-Tb induced a slightly significant increase of COX-2 expression, indicating a minimal interference of these NP on cell metabolism. On the other hand, the challenge of hOB cells with soluble DF, a potent competitive antagonist of COX-2, did not alter the induction of COX-2 expression evoked by inflammatory stimuli. This observation was also confirmed in the cells treated with cAp-Tb-DF 50 µg/mL. Soluble DF 2.5 µg/mL concentration was chosen to pair the amount released from cAp-Tb-DF at 50 µg/mL, as described above. These data were related to prostaglandin-E_2_ release in order to show any possible interaction on COX-2 mRNA expression levels.

### 3.8. DF-Loaded Nanoparticles Inhibit the Release of PGE_2_ from Osteoblasts Treated for Inflammation

**Figure 13:**
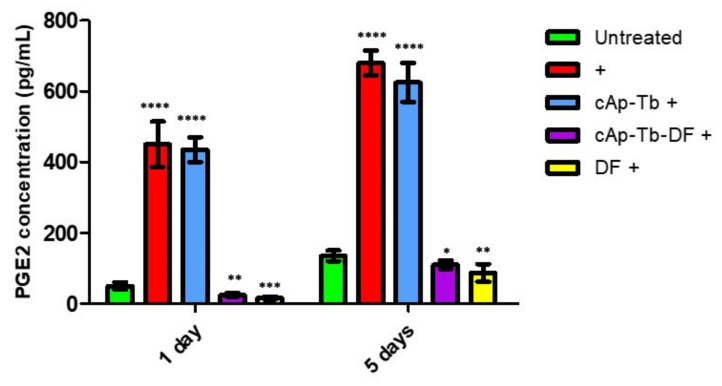
Effect of cAp-Tb-DF NPs on PGE2 released from differentiated hOBs treated with inflammatory cytokine mixture (IL-1β, TNF-α, IFN-γ; +). After 2 days, the different cAp (50 µg/mL) or DF (at comparable amount) were added and cells were incubated for further 1 day or 5 days. (see MM section for details). Data represent means ± sd of three independent experiments performed in triplicate and statistical analyses were carried on using One-way ANOVA, with Bonferroni comparison test. For statistical analysis all data were compared to control untreated samples and the effect of DF was determined vs. the inflammatory treatment. Statistical significance (* *p* < 0.01, ** *p* < 0.005, *** *p* < 0.001, **** *p* < 0.0005).

The concentration of PGE2 in the culture medium was measured on days 1 and 5 using a PGE metabolite assay kit, which converts all major PGE_2_ metabolites in a single stable derivative, thus making more reliable the evaluation of PGE_2_ production. The amount of 50 µg/mL NPs was chosen for these experiments, since 100 µg/mL decreased cell viability, although not significantly. This amount was the best compromise for loading a significant amount of DF, and in the meantime it was fully cytocompatible. PGE_2_ was constitutively released from hOB induced from mesenchymal stem cells at a measurable level (Figure 13: 50 ± 10 pg/mL), which was increased 9 fold (450 ± 64 pg/mL) 1 day after the inflammation treatment. The addition of cAp-Tb-DF to osteoblasts treated for inflammantion reduced the amount of the released PGE_2_ to about 4% (25 ± 5 pg/mL) of the amount released upon the inflammatory treatment, at levels even below the amount of the basally produced PGE2. cAp-Tb NPs did not show a significant inhibitory effect (435 ± 35 pg/mL), while soluble DF at comparable concentrations reduced PGE_2_ release at about the same levels observed for DF loaded on NPs (16 ± 4 pg/mL). Thus, DF treatment reduced of >95% the amount of PGE2 released 1 day after the inflammatory treatment.

When the levels of the released PGE_2_ were measured after 5 day treatments, similar results were observed, although all the values were increased. In this case the amount of constitutively released PGE_2_ was 135 ± 15 pg/mL, which was increased about 5 times by the inflammatory treatment (680 ± 35 pg/mL), reaching even higher levels than those observed after 1 day. Coincubation with cAp-Tb-DF, as well as with soluble DF, resulted in a significant inhibition of cytokine-induced PGE2 production (110 ± 12 and 87 ± 25, respectively), with a reduction around 85%.

## 4. Discussion

cAp NPs are efficient tools for delivering biologically active molecules that might find applications in theranostics, especially in the field of oncology [59,60]. Herein we have prepared cAp and cAp-Tb nanoparticles and investigated their loading/release ability of DF, a potent non-steroidal antiinflammatory drug, using both nanocarriers in different conditions, and we have analyzed their cytocompatibility as well as their biological activities in an in vitro human osteoblast inflammation model. Indeed, today bone tissue pathologies, such as osteoporosis, osteoarthritis, rheumatoid arthritis represent important health problems with considerable socio-economic burden, linked to the general population aging [8,9] and are all characterized by a clinical condition of inflammation.

As a result of the study, we have determined that cAp and cAp-Tb NPs -DF adsorption isotherms follow the Langmuir-Freundlich model at the two temperatures analysed of 25 °C and 37 °C. Our data indicates that the adsorption is heterogeneous and takes place in multilayers, with a finite thickness. The *K_LF_* constants at 25 °C are higher than at 37 °C, for both NPs, thus showing a higher affinity of the DF molecules for the adsorbent at room temperature. Moreover, at 25 °C the adsorption is cooperative (*r* > 1) indicating that the binding of one molecule facilitates the adsorption of the next one, and that two or more molecules can even be adsorbed together. On the contrary, at 37 °C the adsorption is non-cooperative, indicating that the adsorption of one molecule is not facilitated by the adsorption of the previous one. The maximum adsorbed amounts *Q_max_*, however, are greater at 37 °C, although they do not exceed the 0.063 mg/mg adsorbed on cAp-Tb for an initial DF concentration in the solution of 0.45 mg/mL. The influence of the temperature on the adsorption processes, in general, is well documented [51]. Adsorption is an exothermic phenomenon, and thus an increase in temperature leads to a decrease in the adsorption capacity, which results in a lower *K_LF_*, but, at the same time, it favours the solvate-solvate interaction so that the outermost layers attract a greater number of molecules.

Adsorption is influenced also by the pH, especially when the adsorbents are ionic salts, since it determines their surface speciation, i.e., the concentration of cationic, anionic and neutral surface species (in apatite the species > Ca^δ+^ and >PO_x_^δ−^) and, therefore, the net surface charge and the value and sign of the ζ-potential of NP colloidal suspensions. In this work the adsorption experiments were carried out on NPs coated with citrate at pH 7.4. At this pH, the ζ-potentials of cAp and cAp-Tb nanoparticle suspensions are ~ −5 mV and ~20 mV, respectively (Figure 3), indicating that in both cases the surface is negatively charged. The net negative charge is due to the citrate, which is a tricarboxylic acid, whose pKs are pK_1_ = 3.128, pK_2_ = 4.761, and pK_3_ = 6.396. At pH 7.4, citrate would be electrostatically adsorbed on the apatite surface with one or two carboxylates (-COO^−^), leaving the third deprotonated carboxylate exposed towards the solution [48]. In cAp-Tb, some of the surface species would be >Tb^δ+^ atoms, which would mediate a slightly stronger electrostatic adsorption of citrate, resulting in more negatively charged nanoparticle (more negative ζ-potential). After adsorbing DF at pH 7.4 (25 °C), either at 0.3 mg/mL (Q_ads_ = 0.02304 mg/mg), or at 0.45 mg/mL (Q_ads_ = 0.04246 mg/mg) concentrations, both cAp suspensions are stable (ζ-potential very different from 0), although in different pH intervals, as shown in the graphs ofζ-potential versus pH (Figure 3a). In the first case, this is in the range 7 < pH ≤ 9 and to a lesser extent in the interval 4 < pH < 7, while in the second it is in the interval 6 < pH ≤ 8 and to a lesser extent in 4 <pH ≤ 6. Comparing the ζ-potential at pH 7, before and after the adsorption of DF, the values went from −5.0 mV, to −6.9 mV in the first case and to −12.5 mV in the second one. A possible explanation is that the adsorption of Na^+^ and H^+^ on the freely exposed -COO^−^ groups of citrates, which partially neutralizes the negative charge, is followed by the adsorption of DF molecules through the dichloro phenyl ring, leaving the acetate group of the second phenyl exposed toward the solution, whose number would increase for cAp-DF loaded at 0.45 mg/mL (Q_ads_= 0.04246 mg/mg). The multilayer could be produced by π-π interaction between aromatic rings of different DF molecules. In the case of cAp-Tb-DF loaded with the 0.3 mg/mL DF solution (Figure 3b, Q_ads_ = 0.02680 mg/mg), the suspension is quite stable in the range 6 < pH ≤ 8, and unstable at pHs lower and higher than these, since the ζ-potential is around 0. The suspension of cAp-Tb-DF particles loaded with the 0.45 mg/mL solution (Q_ads_ = 0.04435 mg/mg) shows a slight stability in the range 5 ≤ pH ≤ 7, and instability at lower and higher pHs, with ζ-potential values close to 0 (Figure 3b). Comparing at pH 7, the values went from −19.0 mV for the unloaded particles to −10.6 mV and −2.05, for the DF-loaded ones, respectively. The presence of >Tb^δ+^ atoms on the surface of cAp-Tb nanoparticles influenced both citrate and DF binding differently to what observed for the cAp nanoparticles. Thus, DF showed almost similar affinity for the citrate-coated nanoparticles (similar *K_LF_* value), but a lower *r* value, and outermost DF layers were less negatively charged.

Concerning the release trials, a slightly higher amount of drug was found to be released at acidic pH and at 37 °C. This can be explained on the following basis. Since the medium is acidic (pH = 5.2) and DF is a weak acid (pKa = 3.99), its non-ionized form is still abundant and this favours the release of the molecules from the outermost layers in this medium, which would be more loosely linked.

Comparing the results of this work with those of the adsorption of a chemotherapeutic drug (doxorubicin) on cAp previously performed [50], we observed that at 37 °C the *K_LF_* values for DF (*K_LF_*cAp = 0.0572 and K_LF_ cAp-Tb = 0.651) are lower than for doxorubicin (*K_LF_*cAp = 8). This indicates that doxorubicin is more strongly attracted to apatite than DF, since the molecule is positively charged and interacts electrostatically with the free carboxylates of the citrate molecules.

Other studies of DF adsorption have been reported in the literature. In particular, DF was adsorbed on activated carbon at pH 4 at 80 °C, and in this case the adsorption followed the Langmuir model [61], and on bentonite at neutral pH and temperatures from 10 to 77 °C [62]. Nanocarriers of different composition have also been studied for the delivery of DF for biomedical applications, such as polysaccharides [63], poly(ε-caprolactone) micelles [64,65], magnetic NPs [66]. Concerning the adsorption of DF on any form of apatite as adsorbent, it was recently reported the adsorption on amino hydroxyapatite/chitosan/glutaraldehyde hybrids composites at pH 6 [67], and the release of DF from injectable CaP-loaded systems to treat inflammation [68], but these authors did not study the adsorption isotherms, nor used luminescent apatites, therefore we cannot compare our results. In line with what we reported here (see beyond), in all these cases the compositions showed an anti-inflammatory activity on inflammation in vitro and in vivo experimental models.

In this work, Tb^3+^ was incorporated to provide the nanocarrier with a luminescence signal which can be measured to determine where the particles are located for imaging applications [29,55]. The excitation and emission spectra of NPs dispersed in aqueous media at pH 5.2 or pH 7.4 and at different temperatures (25, 37 and 40 °C) were similar. In view of possible biological applications of the system the optimum λ_exc_ and λ_em_ of solid particles suggested are 350 nm and 545 nm, respectively. The luminescence results of the cAp-Tb-DF conjugated nanoparticles show that R.L.I. is higher at pH 5.2 than at pH 7.4, and the luminescence lifetime higher at pH 7.4 than at 5.2, and that neither the concentration of DF nor the temperature affect the R.L.I. and the luminescence lifetime significantly. Thus, this signal cannot be used to determine if DF is released or not. On the other side, the luminescence emission of these particles could be used to determine pH-changes, which are associated to the inflammatory processes. These NPs could be used in the context of inflammation, where local increases of temperature are observed, also because luminescence emission of these NPs does not change between 25 and 40 °C.

When tested on cells, all NPs displayed high cytocompatibility on two human osteosarcoma cell lines after 3 day incubation. These NPs were cytocompabible after 7 days of incubation also on human osteoblasts, both primary, which were obtained from explants of human trabecular bone fragments, and differentiated with an appropriate protocol from human mesenchymal stem cells. These data are in line with previous ones, reporting the properties of other lanthanide-doped nanocrystals [27,69]. The absence of toxicity after long incubation time allowed us to do experiments lasting up to 5 days to assess the effects of the DF loaded on Tb-doped NPs. NPs loaded with the highest amounts of DF (0.45 mg/mg NP) released enough soluble DF (0.046 mg/mg NP) to promote an anti-inflammatory effect in osteoblasts. We adopted a model where a cytokine mixture induced a significant inflammatory response in these cells monitored by the quantification of the cellular release of PGE_2_, an important inflammatory mediator produced after COX-2 enzyme activation [70]. To confirm that these nanoparticles do not interfere with the transcription of this enzyme, we performed quantitative real time PCR for determining COX-2 mRNA levels. Our data show that these nanocarriers do not influence cell metabolism at a transcription level. These data were also confirmed by the high levels of COX-2 mRNA expression observed after the treatment with DF-loaded cAp-Tb or soluble DF in the inflammation cell model which were comparable to the levels observed for the inflammation stimuli treatment. Thus, the significant reduction in the PG2_2_ release that we observed after the treatment of the inflamed cells with DF loaded cAp-Tb could be attributed specifically to the ability of cAp-Tb to release DF.

These results show the potential of cAp nanoparticles as nanocarriers for loading and controlled release of DF, and of cAp-Tb nanoparticles as luminescent nanoprobes with diagnostic, as well as therapeutic (theranostic) applications in pathological conditions.

## 5. Conclusions

We have studied the loading/release ability of DF on biomimetic cAp and cAp-Tb. The adsorption of the drug in both apatites has shown that, both at 25 °C and at 37 °C, follows the Langmuir-Freundlich model, giving rise to the formation of multilayers. The *K_LF_* constants at 25 °C (*K_LF_* cAp = 2.77 and *K_LF_* cAp-Tb = 2.98) are greater than at 37 °C (*K_LF_* cAp = 0.0572, and *K_LF_* cAp-Tb = 0.651), reflecting a higher affinity for the molecules by the nanoparticles at 25 °C, and in particular towards cAp-Tb. Furthermore, in both cases the adsorption is cooperative.

The release of DF from the nanoparticles is favoured when it occurs at an acidic pH (5.2) and at a temperature of 37 °C, releasing 2% more of the drug than in a neutral medium. This data is relevant since the aim of this study is to be able to use biomimetic nanoparticles loaded with DF in pathological conditions, in bone trauma and fractures, in which prolonged inflammatory processes occur with a local increase in temperature and a decrease in the pH of the medium. The luminescence results of the cAp-Tb-DF conjugated nanoparticles show that R.L.I. is higher at pH 5.2 than at pH 7.4, and the luminescence lifetime higher at pH 7.4 than at 5.2, and that neither the concentration of DF nor the temperature affect the R.L.I. and the luminescence lifetime significantly. Luminescence emission of these particles could have biological applications in inflammation, since it can detect pH-changes, not being affected by the raise of temperature, being both conditions associated with the inflammatory process. Concerning cell behaviour, all the nanocarriers studied showed no reduction in osteoblasts cell vitality and in an in vitro model of osteoblasts cytokine–induced inflammation, they affected COX-2 expression and decreased the cellular production of prostaglandin E_2_. These results show the potential of cAp nanoparticles as nanocarriers for loading and controlled release of DF, and of cAp-Tb nanoparticles as luminescent nanoprobes with diagnostic, as well as therapeutic (theranostic) applications in pathological conditions.

## Figures and Tables

**Table 1:** Regression coefficients and parameters *K_LF_*, *r* and *Q_max_* determined from Langmuir-Freundlich model.

Substrate, *T* (°C)	R^2^	*K_LF_*	*r*	*Q_max_* (mg/mg)	Adsorption Type
cAp, 25 °C	0.9679	2.77	1.794	0.04648	Cooperative, high affinity
cAp, 37 °C	0.9709	0.05	0.5536	0.05067	Non-cooperative, low affinity
cAp-Tb, 25 °C	0.9503	2.98	1.0973	0.05411	Cooperative, high affinity
cAp-Tb, 37 °C	0.9482	0.65	0.3751	0.06306	Non-cooperative, low affinity

**Table 2:** Maximum released amount of DF per unit mass of adsorbent (*Q_D,max_*, mg/mg) and *Dr* at pHs 7.4 and 5.2 for cAp-DF and cAp-Tb-DF nanoassemblies.

Parameter	cAp-DF, 25 °C, pH = 7.4	cAp-DF, 37 °C, pH = 7.4	cAp-Tb-DF, 25 °C, pH = 7.4	cAp-Tb-DF, 37 °C, pH = 7.4	cAp-DF, 25 °C, pH = 5.2	cAp-DF, 37 °C, pH = 5.2	cAp-Tb-DF, 25 °C, pH = 5.2	cAp-Tb DF, 37 °C, pH = 5.2
*Q_Dmax_* (mg/mg)	0.01128	0.01177	0.01007	0.01066	0.01048	0.01300	0.01073	0.01185
*Dr* (%wt)	24.26	23.24	18.55	16.90	23.33	25.66	19.84	18.79

## Data Availability

Not applicable.

## Supplementary Materials

The following figures are available online at www.mdpi.com/article/10.3390/nano12030562/s1. Figure S1: X-ray diffraction patterns of cAp and cAp-Tb prepared by thermal decomplexing method, Figure S2: (a) FTIR and (b) Raman spectra of cAp and cAp-Tb prepared by thermal decomplexing method, Figure S3: (a) PSD in volume and (b) cumulative oversize PSD of cAp and cAp-Tb. Figure S4: Evolution of the adsorbed amount of DF with time on cAp (a) and cAp-Tb (b). Figure S5: Excitation (dashed lines) and emission (solid lines) uncorrected spectra of samples containing different DF concentrations dispersed in a pH = 5.2 aqueous suspension at 0.5 mg/mL and 25 °C. Figure S6: Excitation (dashed lines) and emission (solid lines) uncorrected spectra of samples containing different DF concentrations dispersed in a pH = 5.2 aqueous suspension at 0.5 mg/mL and 40 °C. Figure S7: Excitation (dashed lines) and emission (solid lines) uncorrected spectra of samples containing different DF concentrations dispersed in a pH = 7.4 aqueous suspension at 0.5 mg/mL and 25 °C. Figure S8: Excitation (dashed lines) and emission (solid lines) uncorrected spectra of samples containing different DF concentrations dispersed in a pH = 7.4 aqueous suspension at 0.5 mg/mL and 37 °C. Figure S9: Excitation (dashed lines) and emission (solid lines) uncorrected spectra of samples containing different DF concentrations dispersed in a pH = 7.4 aqueous suspension at 0.5 mg/mL and 40 °C. Figure S10: Luminescence decay curves at different DF concentrations of 0.5 mg/mL suspended particles at 25 °C and pH 5.2. λ_exc/em_ = 350/545 nm, t_d_ = 120 µs, t_g_ = 0.01 ms, slitwidth_exc/em_ = 20/20 nm and detector voltage 900 v. Figure S11: Luminescence decay curves at different DF concentrations of 0.5 mg/mL suspended particles at 37 °C and pH 5.2. λ_exc/em_ = 350/545 nm, t_d_ = 120µs, t_g_ = 0.01 ms, slitwidth_exc/em_ = 20/20 nm and detector voltage 900 v. Figure S12: Luminescence decay curves at different DF concentrations of 0.5 mg/mL suspended particles at 40 °C and pH 5.2. λ_exc/em_ = 350/545 nm, t_d_ = 120 µs, t_g_ = 0.01 ms, slitwidth_exc/em_ = 20/20 nm and detector voltage 900 v. Figure S13: Luminescence decay curves at different DF concentrations of 0.5 mg/mL suspended particles at 25 °C and pH 7.4. λ_exc/em_ = 350/545 nm, t_d_ = 120 µs, t_g_ = 0.01 ms, slitwidth_exc/em_ = 20/20 nm and detector voltage 900 v. Figure S14: Luminescence decay curves at different DF concentrations of 0.5 mg/mL suspended particles at 37 °C and pH 7.4. λ_exc/em_ = 350/545 nm, t_d_ = 120 µs, t_g_ = 0.01 ms, slitwidth_exc/em_ = 20/20 nm and detector voltage 900 v. Figure S15: Luminescence decay curves at different DF concentrations of 0.5 mg/mL suspended particles at 40 °C and pH 7.4. λ_exc/em_ = 350/545 nm, t_d_ = 120 µs, t_g_ = 0.01 ms, slitwidth_exc/em_ = 20/20 nm and detector voltage 900 v. Figure S16: Viability of MG-63 cells (a) and of U-2OS cells (b) incubated with cApTb-DF particles loaded with different DF concentration, ranging from 0.1 to 0.45 mg/mg NP, for three days. Figure S17: Osteoblasts differentiated from human mesenchymal stem cells express alkaline phosphatase.
