# Supplementary material for: Biomimetic Citrate-Coated Luminescent Apatite Nanoplatforms for Diclofenac Delivery in Inflammatory Environments
Source: Nanomaterials (Basel). 2022 Feb 6;12(3):562. doi: 10.3390/nano12030562 (PMC8838995; doi:10.3390/nano12030562)
Supplement: Supplementary file 1 [file nanomaterials-12-00562-s001.zip › nanomaterials-1560154-supplementary.pdf]

# Supplementary Materials

# Biomimetic Citrate-Coated Luminescent Apatite Nanoplatforms for Diclofenac Delivery in Inflammatory Environments

**Sandra Maria Cano Plá [1,†], Annarita D'Urso [2,†], Jorge Fernando Fernández-Sánchez [3], Donato Colangelo [2], Duane Choquesillo-Lazarte [1], Riccardo Ferracini [4,5], Michela Bosetti [6], Maria Prat [2,7,8,9,*] and Jaime Gómez-Morales [1,*]**

1. Laboratorio de Estudios Cristalográficos, IACT, CSIC-UGR, Avda. Las Palmeras, n° 4, E-18100 Armilla, Granada, Spain; sandra@lec.csic.es (S.M.C.P.); duane.choquesillo@csic.es (D.C.-L.)
2. Dipartimento di Scienze della Salute, Università del Piemonte Orientale, "A. Avogadro" Via Solaroli, 17, 28100 Novara, Italy; annarita.durso@med.uniupo.it (A.D.); donato.colangelo@med.uniupo.it (D.C.)
3. Department of Analytical Chemistry, Faculty of Sciences, University of Granada, Avda. Fuentenueva s/n, 18071 Granada, Spain; jffernan@ugr.es (J.F.F.-S.)
4. Dipartimento di Scienze Chirurgiche e Diagnostiche Integrate, Università di Genova, Viale Benedetto XV 6, 16132 Genova, Italy; ferracini@edu.unige.it
5. Ospedale Koelliker, Corso Galileo Ferraris, 247/255, 10134 Torino, Italy
6. Dipartimento di Scienze del Farmaco, Università del Piemonte Orientale "A. Avogadro", Via Bovio 4, 28100 Novara, Italy; michela.bosetti@uniupo.it
7. Centro di Biotecnologie per la Ricerca Medica Applicata (BRMA), Via Solaroli 17, 28100 Novara, Italy
8. Consorzio Interuniversitario per Biotecnologie (CIB), Località Padriciano 99, 34149 Area di Ricerca, Trieste, Italy
9. Consorzio Interuniversitario Nazionale per la Scienza e Tecnologia dei Materiali (INSTM), Via Giuseppe Giusti, 9, 50121 Firenze, Italy.

* Correspondence: maria.prat@med.uniupo.it (M.P.); jaime@lec.csic.es (J.G.-M.); Tel.: +39-0321660662 (M.P.); 34-958525020 (J.G.-M.)

† These authors equally contributed to this work.

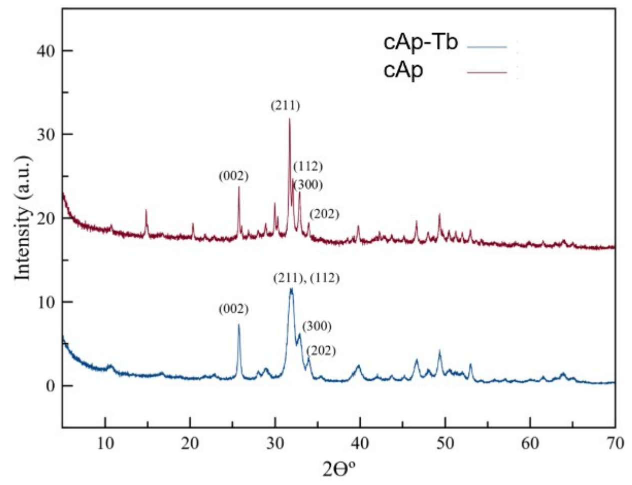


**Figure S1.** X-ray diffraction patterns of cAp and cAp-Tb prepared by thermal decomplexing method.

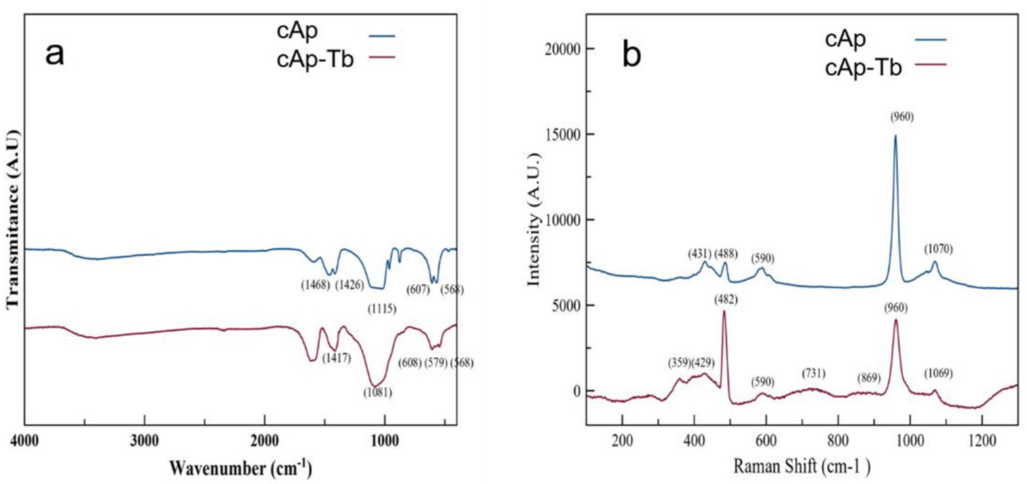


**Figure S2.** a) FTIR and b) Raman spectra of cAp and cAp-Tb prepared by thermal decomplexing method.

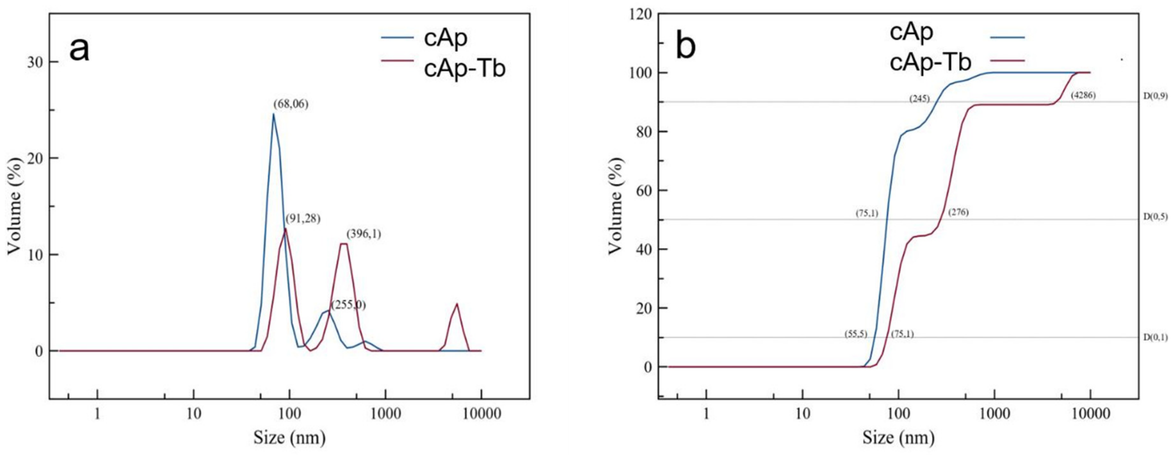


**Figure S3.** a) PSD in volume and b) cumulative oversize PSD of cAp and cAp-Tb.

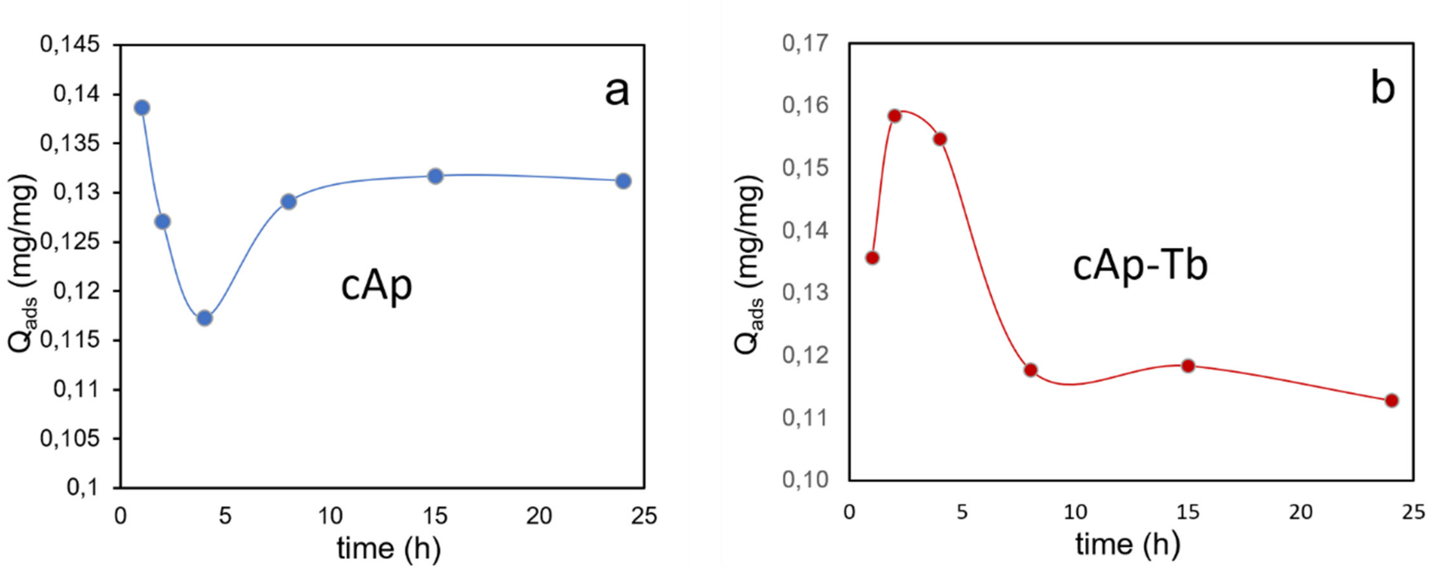


**Figure S4.** Evolution of the adsorbed amount of DF with time on cAp (a) and cAp-Tb (b).

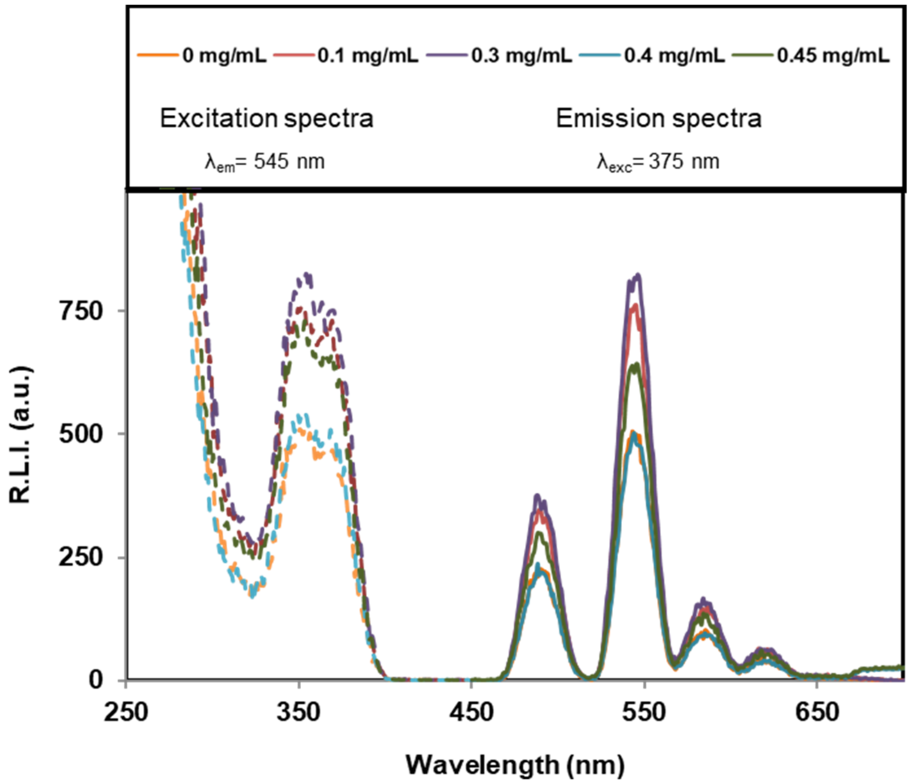


**Figure S5.** Excitation (dashed lines) and emission (solid lines) uncorrected spectra of samples containing differentDF concentrations dispersed in a pH=5.2 aqueous suspension at 0.5mg/mL and 25 °C.

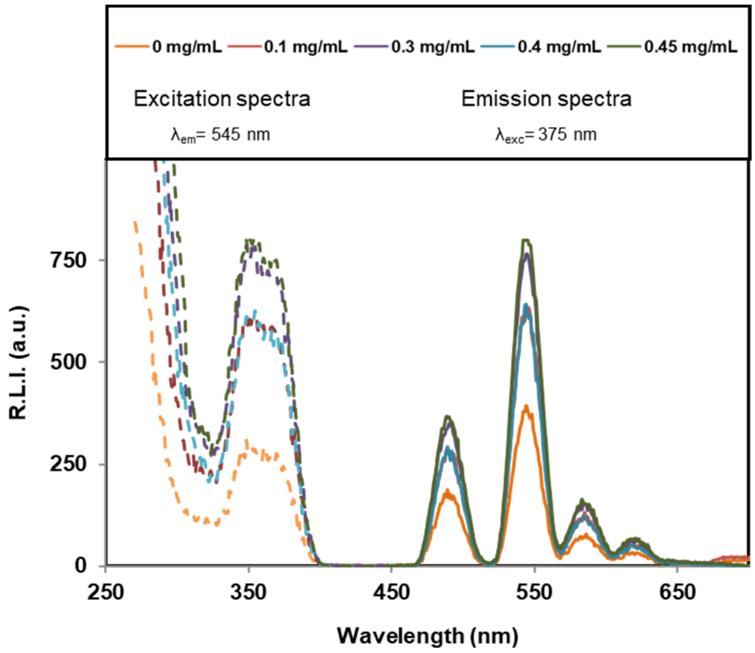


**Figure S6.** Excitation (dashed lines) and emission (solid lines) uncorrected spectra of samples containingdifferent DF concentrations dispersed in a pH=5.2 aqueous suspension at 0.5mg/mL and 40 °C.

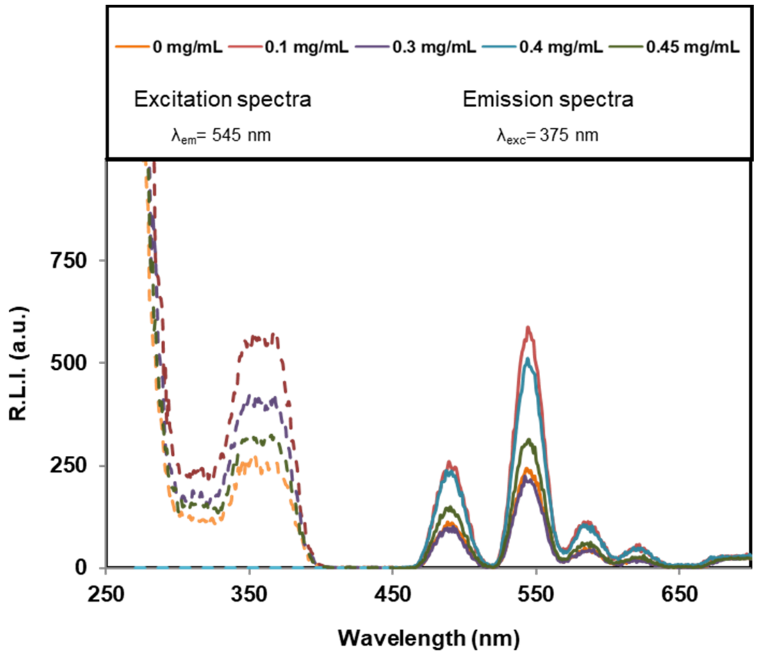


**Figure S7**. Excitation (dashed lines) and emission (solid lines) uncorrected spectra of samples containingdifferent DF concentrations dispersed in a pH=7.4 aqueous suspension at 0.5mg/mL and 25 °C.

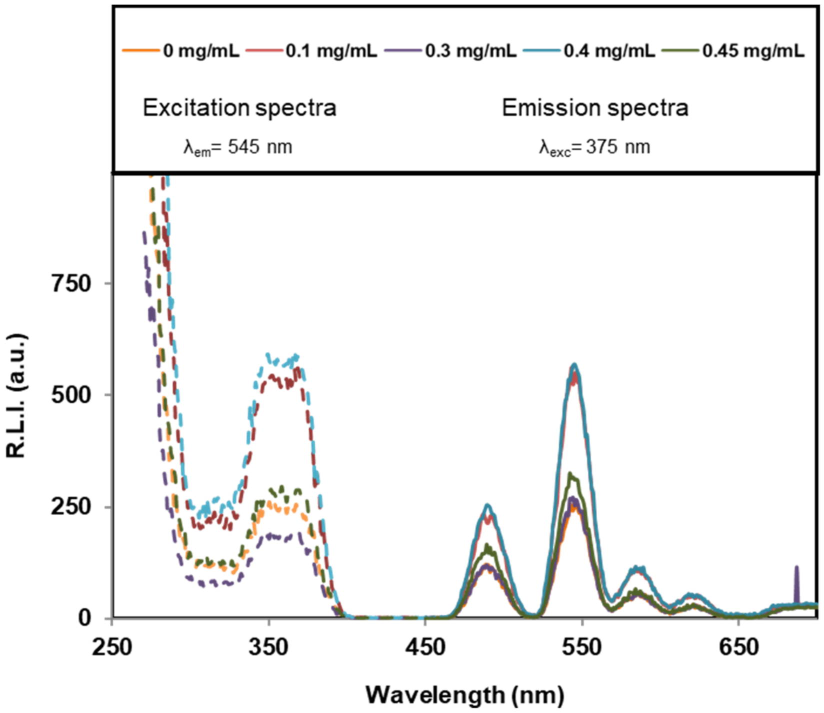


**Figure S8.** Excitation (dashed lines) and emission (solid lines) uncorrected spectra of samples containingdifferent DF concentrations dispersed in a pH=7.4 aqueous suspension at 0.5mg/mL and 37 °C.

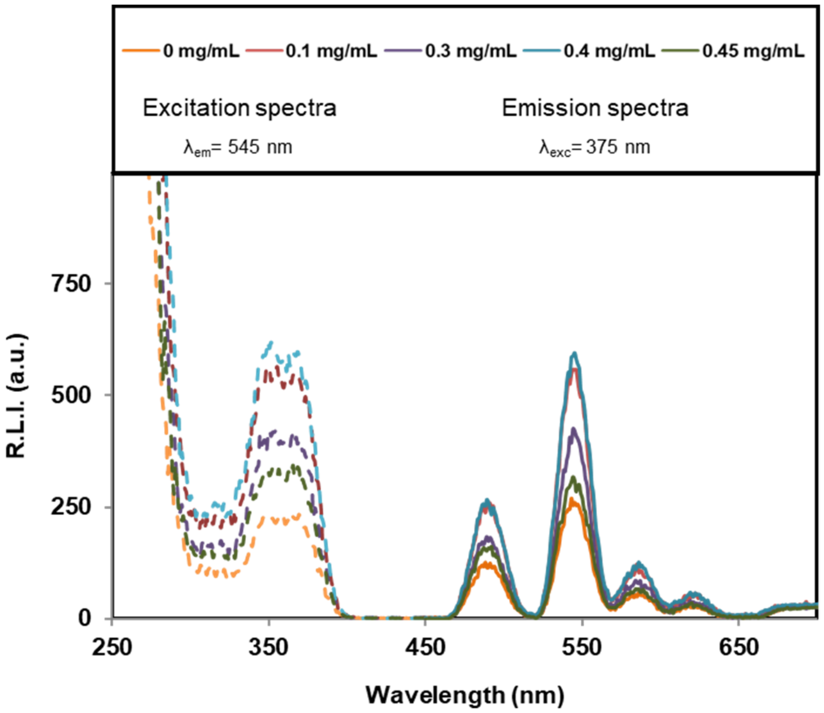


**Figure S9.** Excitation (dashed lines) and emission (solid lines) uncorrected spectra of samples containing different DF concentrations dispersed in a pH=7.4 aqueous suspension at 0.5mg/mL and 40 °C.

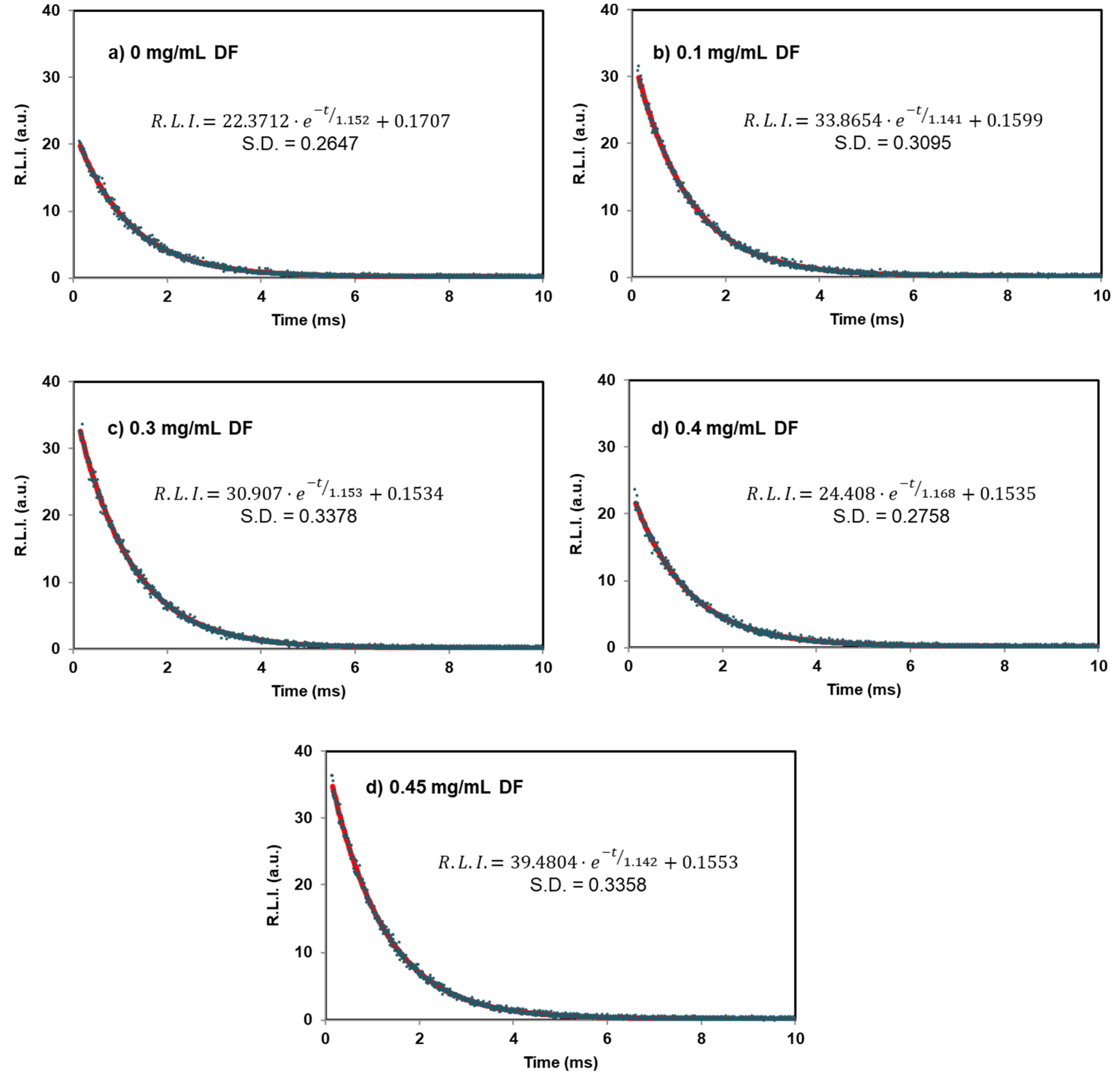


**Figure S10**. Luminescence decay curves at different DF concentrations of 0.5 mg/mL suspended particles at 25 °C and pH 5.2. $\lambda_{exc/em}$=350/545 nm, $t_d$=120µs, $t_g$=0.01 ms, slit-width$_{exc/em}$=20/20 nm and detector voltage 900v.

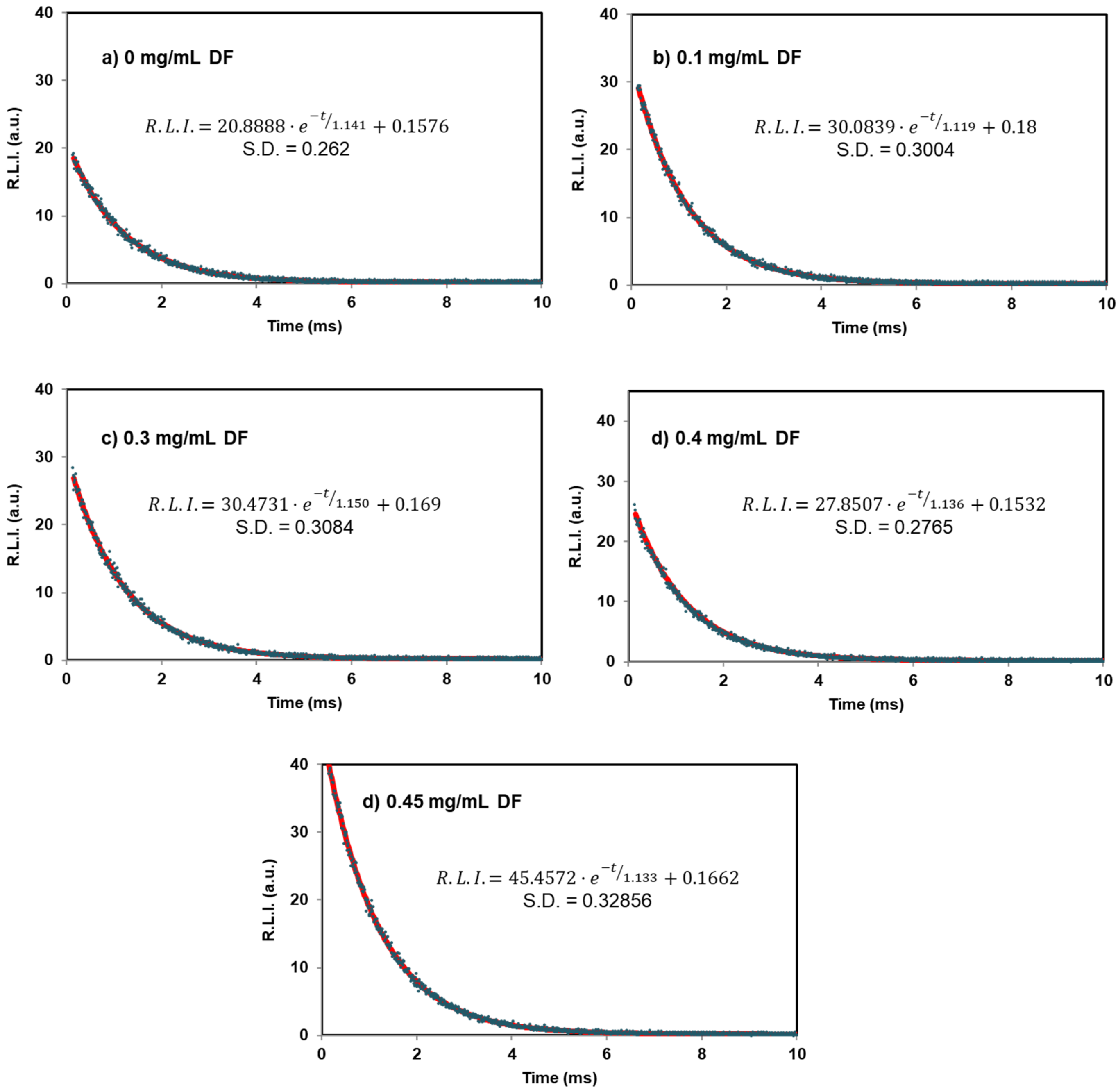


**Figure S11.** Luminescence decay curves at different DF concentrations of 0.5 mg/mL suspended particles at 37 °C and pH 5.2. $\lambda_{exc/em}$=350/545 nm, $t_d$=120μs, $t_g$=0.01 ms, slitwidth$_{exc/em}$=20/20 nm and detector voltage 900v.

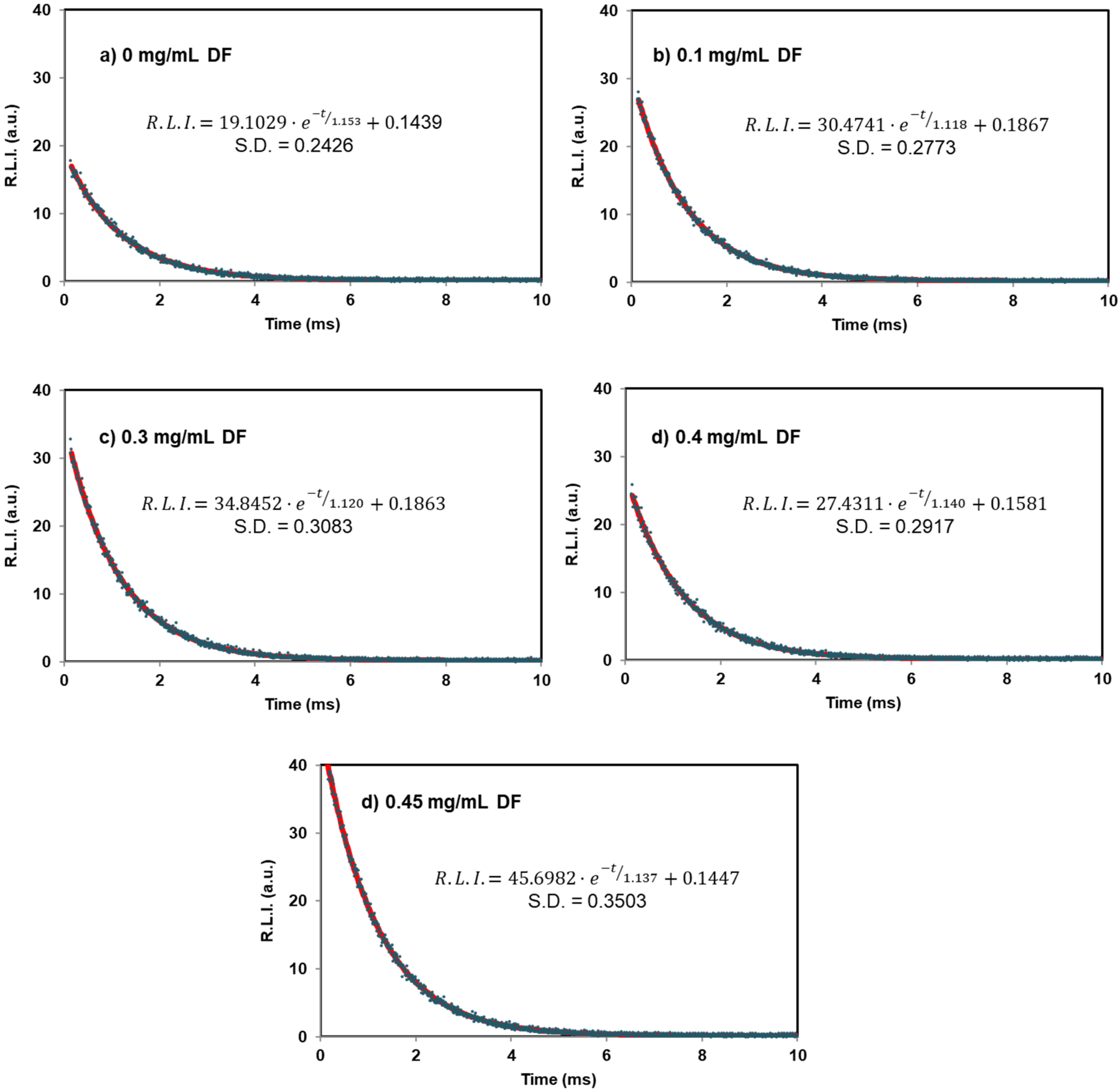


**Figure S12.** Luminescence decay curves at different DF concentrations of 0.5 mg/mL suspended particles at 40 °C and pH 5.2. $\lambda_{exc/em}$=350/545 nm, $t_d$=120µs, $t_g$=0.01 ms, slitwidth$_{exc/em}$=20/20 nm and detector voltage 900v.

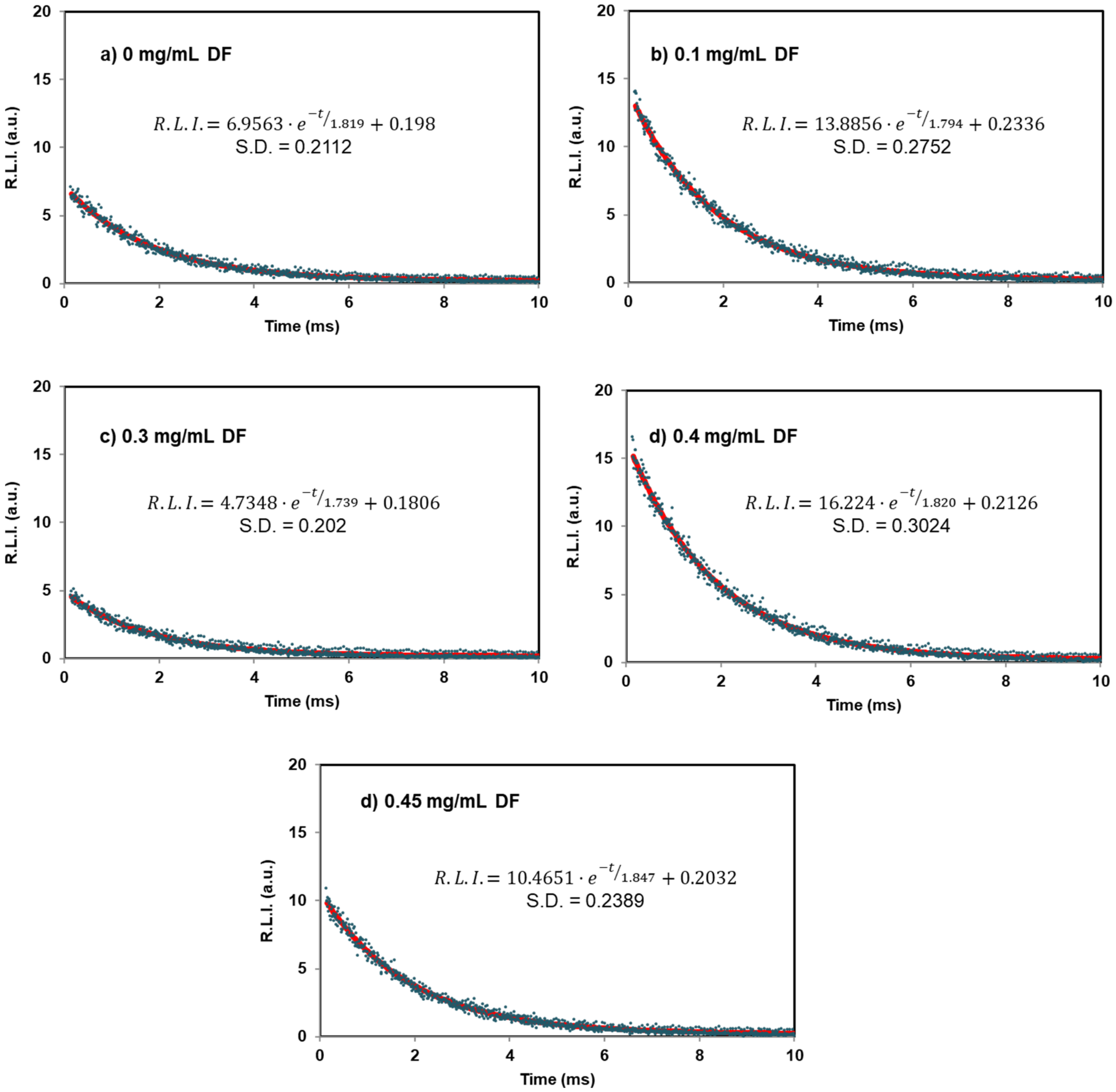


**Figure S13.** Luminescence decay curves at different DF concentrations of 0.5 mg/mL suspended particles at 25 °C and pH 7.4. $\lambda_{exc/em}$=350/545 nm, $t_d$=120μs, $t_g$=0.01 ms, slitwidth$_{exc/em}$=20/20 nm and detector voltage 900v.

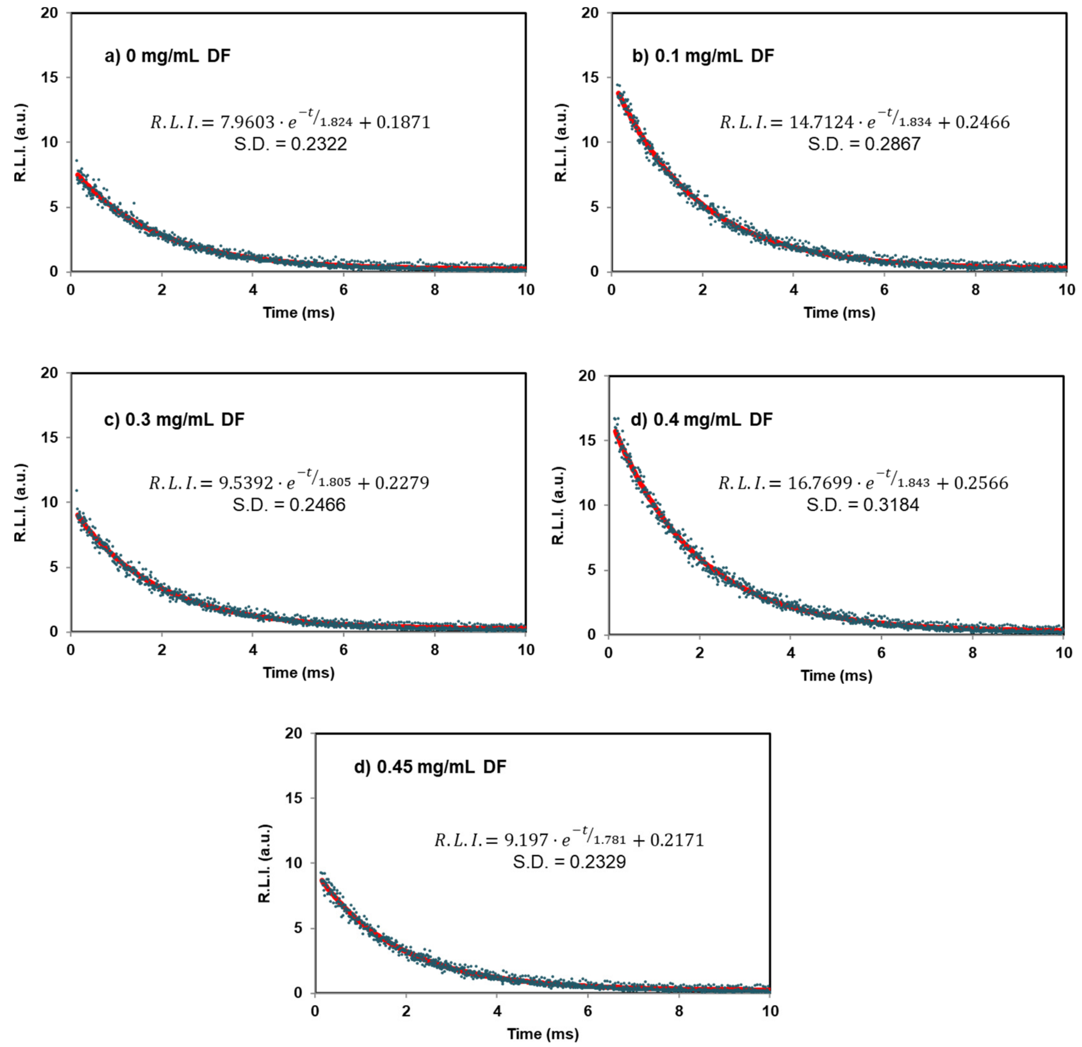


**Figure S14.** Luminescence decay curves at different DF concentrations of 0.5 mg/mL suspended particles at 37 °C and pH 7.4. $\lambda_{exc/em}$=350/545 nm, $t_d$=120µs, $t_g$=0.01 ms, $slitwidth_{exc/em}$=20/20 nm and detector voltage 900v.

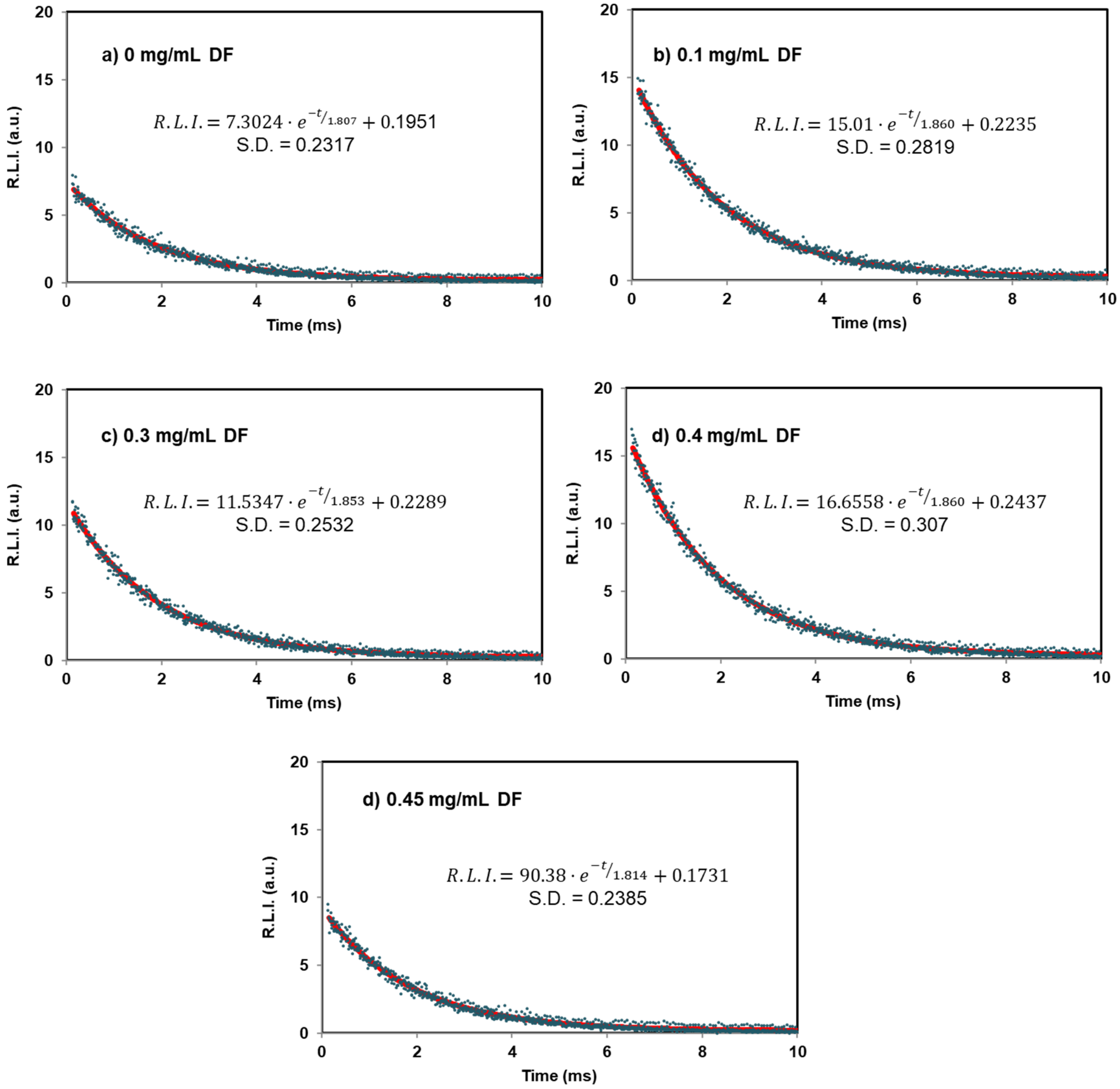


**Figure S15.** Luminescence decay curves at different DF concentrations of 0.5 mg/mL suspended particles at 40 °C and pH 7.4. $\lambda_{exc/em}$=350/545 nm, $t_d$=120μs, $t_g$=0.01 ms, slitwidth$_{exc/em}$=20/20 nm and detector voltage 900v.

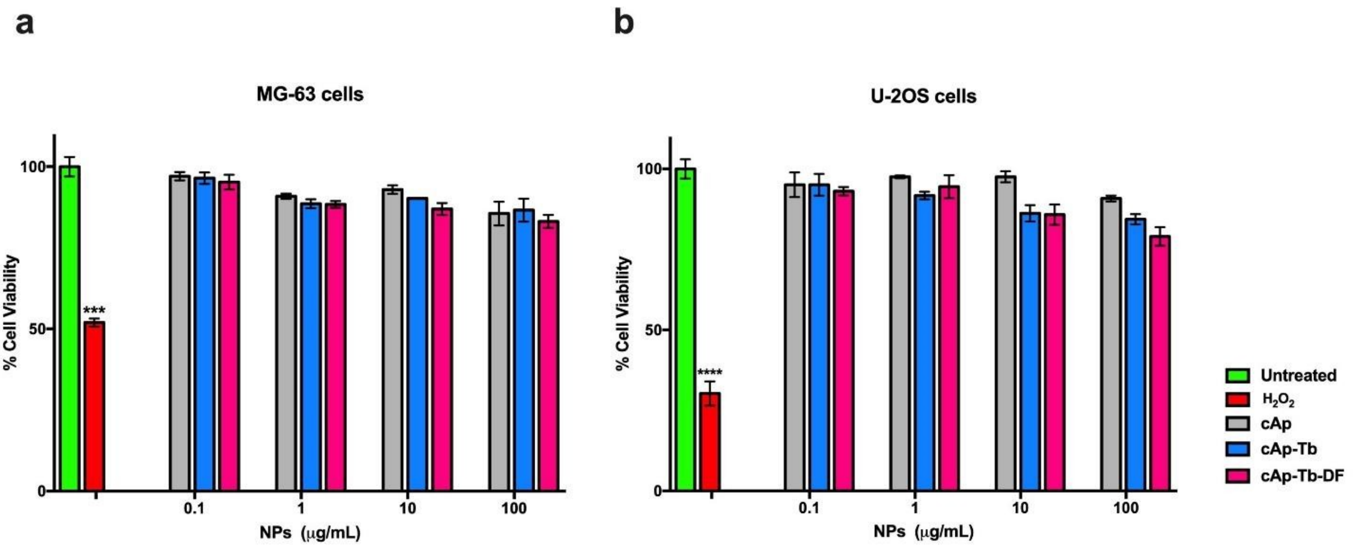


**Figure S16.** Viability of MG-63 cells (a) and of U-2OS cells (b) incubated with different concentrations of cApTb nanoparticles loaded with 0.45 mg/mg DF for three days. Viability was assessed in MTT assays. Datarepresent means ± sd of three independent experiments performed in triplicate and statistical analyses were carried on using One-way ANOVA, with Bonferroni comparison test. For statistical analysis all data were compared to untreated samples and only samples treated with 1μM $H_2O_2$ displayed statistically significant difference (*** $p \leq 0.001$, **** $p \leq 0.0005$).

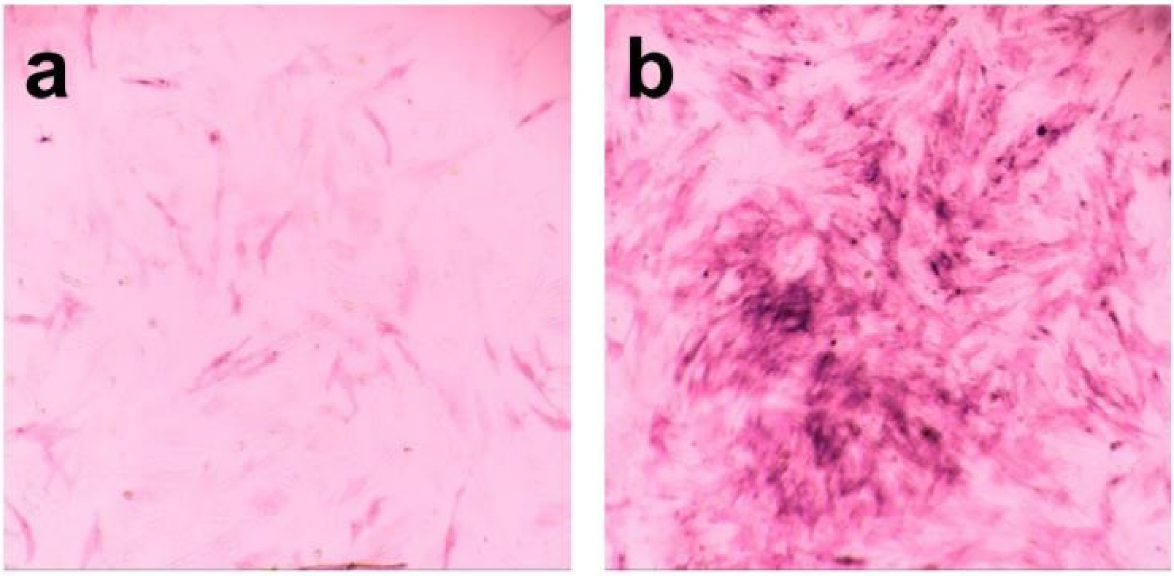


**Figure S17.** Osteoblasts differentiated from human mesenchymal stem cells express alkaline phosphatase. Mesenchymal stem cells obtained from the stromal vascular fraction of lipoaspirates, after enzymatic digestion with Collagenase NB4 and elimination of blood cells, were induced to bone differentiation by incubation with complete Ob medium containing 50 mg/mL ascorbic acid, 10 mM β-glicerophosphate, and 10 nM dexamethasone for 2 weeks, which medium changes every 3-4 days. Cells (125,000 cells/well) were then seeded in 12-well plates with 2 mL medium. After 2 days cells were washed with PBS and fixed with 4% paraformaldehyde for 30 min. The paraformaldehyde was aspirated and 1 mL alkaline phosphatase staining solution was added and left to react for 30 min in the dark at room temperature. The reaction was stopped by washing quickly with distilled water, and images were taken with an an inverted microscope at a20x enlargement. a, untreated mesenchymal stem cells; b, cells treated for osteoblastic differentiation.
